# Comparative transcriptome and proteome analysis reveals a global impact of the nitrogen regulators AreA and AreB on secondary metabolism in *Fusarium fujikuroi*

**DOI:** 10.1371/journal.pone.0176194

**Published:** 2017-04-25

**Authors:** Andreas Pfannmüller, Johannes Leufken, Lena Studt, Caroline B. Michielse, Christian M. K. Sieber, Ulrich Güldener, Susan Hawat, Michael Hippler, Christian Fufezan, Bettina Tudzynski

**Affiliations:** 1 Institute of Biology and Biotechnology of Plants, Molecular Biology and Biotechnology of Fungi, Westfälische Wilhelms-University Münster, Münster, Germany; 2 Institute of Biology and Biotechnology of Plants, Computational Biology, Westfälische Wilhelms-University Münster, Münster, Germany; 3 Division of Microbial Genetics and Pathogen Interaction, Department of Applied Genetics and Cell Biology, Campus-Tulln, BOKU-University of Natural Resources and Life Science, Vienna, Austria; 4 Institute of Bioinformatics and Systems Biology, Helmholtz Zentrum München, German Research Center for Environmental Health (GmbH), Neuherberg, Germany; 5 Department of Genome-oriented Bioinformatics, Wissenschaftszentrum Weihenstephan, Technische Universität München, Freising, Germany; 6 Institute of Biology and Biotechnology of Plants, Plant Biochemistry and Biotechnology, Westfälische Wilhelms-University Münster, Münster, Germany; Soonchunhyang University, REPUBLIC OF KOREA

## Abstract

The biosynthesis of multiple secondary metabolites in the phytopathogenic ascomycete *Fusarium fujikuroi* is strongly affected by nitrogen availability. Here, we present the first genome-wide transcriptome and proteome analysis that compared the wild type and deletion mutants of the two major nitrogen regulators AreA and AreB. We show that AreB acts not simply as an antagonist of AreA counteracting the expression of AreA target genes as suggested based on the yeast model. Both GATA transcription factors affect a large and diverse set of common as well as specific target genes and proteins, acting as activators and repressors. We demonstrate that AreA and AreB are not only involved in fungal nitrogen metabolism, but also in the control of several complex cellular processes like carbon metabolism, transport and secondary metabolism. We show that both GATA transcription factors can be considered as master regulators of secondary metabolism as they affect the expression of more than half of the 47 putative secondary metabolite clusters identified in the genome of *F*. *fujikuroi*. While AreA acts as a positive regulator of many clusters under nitrogen-limiting conditions, AreB is able to activate and repress gene clusters (e.g. bikaverin) under nitrogen limitation and sufficiency. In addition, ChIP analyses revealed that loss of AreA or AreB causes histone modifications at some of the regulated gene clusters.

## Introduction

The ascomcyete *Fusarium fujikuroi* is a notorious plant pathogen, most famous for causing the ‘bakanae’ or ‘foolish seedling’ disease in rice plants [1]. Infected plants display an abnormal elongation of internodes, which is caused by the fungal production of gibberellins (GA), a class of bioactive diterpenoid plant hormones [2–4]. Besides GA, *F*. *fujikuroi* produces a broad spectrum of other secondary metabolites (SM), including the red pigments bikaverin (BIK) and fusarubins (FSR) [5,6], as well as the mycotoxins fusarins (FUS), fusaric acid (FSA), fumonisins (FUM), beauvericin (BEA) and apicidin F (APF), the latter being uniquely produced by *F*. *fujikuroi* [7–15].

The recently sequenced genome of *F*. *fujikuroi* spans 12 chromosomes containing 14,813 predicted gene models and a total of 47 putative SM gene clusters centered around the following biosynthetic key-enzymes: 14 polyketide synthases (PKS), 15 non-ribosomal peptide synthetases (NRPS), 4 PKS/NRPS hybrid enzymes, 2 dimethylallyl tryptophan synthases (DMATS) and 12 terpene cyclases (TC) [8]. Most of these gene clusters (32 out of 47), including the GA cluster, do not contain cluster-specific transcription factors (TFs) suggesting that they are regulated by global TFs depending on the environmental conditions. One crucial environmental cue affecting the expression of many SM clusters in *F*. *fujikuroi* is the nitrogen (N) availability to the fungus. In fact, 30 of the *F*. *fujikuroi* SM gene clusters were found to be regulated by N [8]. Some of them are only expressed under N-sufficient conditions (e.g. FUS, FUB (fusaric acid biosynthesis), APF), while others are subject to nitrogen metabolite repression (NMR) and are therefore only expressed under N-limiting conditions (e.g. GA, FUM, BIK, and FSR) [4–6,8–11]. N-repression of SM has also been described for other fungi, e.g. the biosynthesis of penicillin in *Penicillium chrysogenum*, trichothecenes and fusarielin H in *Fusarium graminearum*, and cephalosporin in *Acremonium chrysogenum* [16–19]. The GA biosynthetic genes in *F*. *fujikuroi* were the first genes shown to be under the control of the major N metabolism regulator AreA [20].

AreA (NIT2 in *Neurospora crassa*) is a GATA-family TF with a Cys_2_Cys_2_ Zinc finger DNA-binding domain preferentially binding to at least two 5’HGATAR DNA sequence motifs within a distance of 30 bp [21–24]. This key regulator mediates de-repression of genes involved in the utilization of alternative N sources in the absence of preferred N sources like glutamine and ammonium [25]. Alternatively, AreA can control expression of genes synergistically with other TFs. The best studied model is the regulation of the nitrate assimilation system in *A*. *nidulans*, which relies on the activities of the two TFs AreA and NirA [26–28]. Here, the activity of AreA opens the chromatin structure at the nitrate utilization gene cluster via histone acetylation thereby enabling binding of the nitrate-activated TF NirA to its target promoters, the nitrate assimilatory genes [28–30]. These findings suggest that AreA can bind directly to promotors of target genes and is able to regulate gene expression by recruitment of other TFs and/or post-translational histone modifications.

Besides AreA, a second GATA TF was shown to be involved in N-dependent gene regulation in *A*. *nidulans* and *P*. *chrysogenum* termed AreB and NreB, respectively [31–35]. In these two fungi, AreB/NreB is generally regarded as the negative counterpart to AreA, acting as a major repressor of AreA-activated N catabolism genes [32,35]. However, the function of AreB appears to be more complex: both AreA and AreB were shown to repress the arginine catabolism genes *agaA* and *otaA* under N-repressing and carbon(C)-limiting conditions [36,37]. Recently, we investigated the role of AreB and its interplay with AreA in *F*. *fujikuroi*, where we could show that the deletion of *AREB* resulted in a general growth reduction on various media, but had no effect on the use of alternative N or C sources [31]. Furthermore, expression analysis of selected N-regulated genes indicated a dynamic regulatory interplay of AreA and AreB: expression of the GA cluster genes was completely lost in Δ*AREA* as well as Δ*AREB*, suggesting a synergistic activation regulation by both TFs. Finally, we showed for the first time that AreA and AreB physically interact in the nucleus under N-limiting conditions [31].

In view of these unexpected and intriguing results we have examined the common, as well as the different, effects of AreA and AreB on gene expression on a genome-wide level in *F*. *fujikuroi* using microarray analysis. Additionally, we compared the abundance of proteins between the wild type (Wt) and Δ*AREA* and Δ*AREB* mutants and investigated the potential effect of *AREA* and *AREB* deletion on histone H3 lysine 9 (H3K9) acetylation profiles around SM gene clusters by chromatin immunoprecipitation (ChIP) analysis. These comprehensive studies demonstrate that both GATA TFs are major regulators of many metabolic processes which may coordinately activate or repress common target genes, but that both of them have also specific functions. Besides regulating alternative N assimilation pathways, both AreA and AreB are global regulators of secondary metabolism. While AreA mainly acts as a positive regulator of many SM gene clusters under N-limiting conditions, AreB activates and represses certain SM genes under N-limiting as well as N-sufficient conditions. The combined transcriptome, proteome and ChIP analyses give insights into their mode of regulation and provide a basis for further studies of the complex N regulation network in *F*. *fujikuroi* and other filamentous fungal species.

## Results

To gain a deeper insight into the regulatory effects of AreA and AreB in *F*. *fujikuroi*, an integrative analysis of the transcriptome and proteome under N-limiting (6 mM glutamine) and N-sufficient (60 mM glutamine) conditions was performed in biological duplicates. Differences between the Wt and mutant strains under either condition were studied at the transcriptome and proteome levels by microarray and high performance liquid chromatography coupled to tandem mass spectrometry (HPLC-MS/MS), respectively.

### Deletion of *AREA* and *AREB* affects the transcription of large gene sets

**Fig 1:**
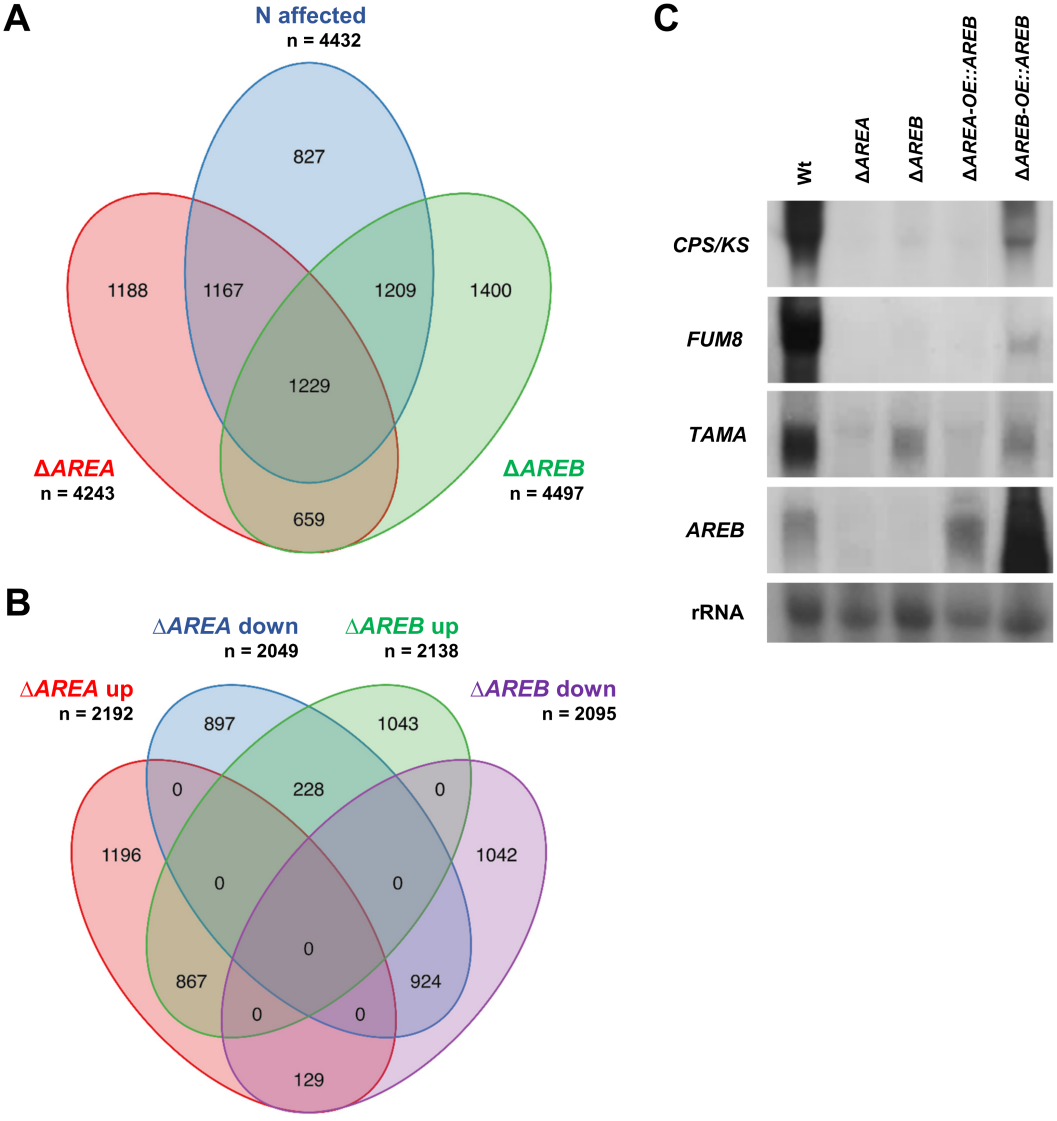
Distribution of genes affected in Δ*AREA* and Δ*AREB*, as well as by nitrogen availability. The *F*. *fujikuroi* Wt and the Δ*AREA* and Δ*AREB* deletion mutants were cultivated for 3 days in ICI liquid cultures with 6 mM glutamine (nitrogen limitation) or 60 mM glutamine (nitrogen sufficiency) as sole nitrogen source. Data is based on microarray analysis. (A) Differentially regulated genes at 60 mM compared to 6 mM glutamine (N affected), as well as in Δ*AREA* and Δ*AREB* compared to Wt. (B) Differentially up-regulated (log_2_FC > 1) and down-regulated (log_2_FC < -1) genes at 6 mM glutamine in Δ*AREA* and Δ*AREB* compared to Wt. (C) Northern blot analysis of SM cluster genes *CPS/KS* (GA) and *FUM8* (FUM) as well as TF genes *TAMA* and *AREB*. The *F*. *fujikuroi* Wt, the Δ*AREA* and Δ*AREB* deletion mutants and two strains expressing *AREB* ectopically under control of a strong, constitutive promoter (Δ*AREA-OE*::*AREB*) were cultivated at 6 mM glutamine for 3 days.

The genome-wide search for AreA- and AreB-dependent genes was performed by use of high quality 126135 K NimbleGen microarrays that were manufactured based on the present genome annotation of *F*. *fujikuroi* IMI58289 [8]. Based on the selection criteria 2-fold change in expression at the 95% confidence interval (False Discovery Rate < 0.05), 4,432 of the 14,813 annotated genes were affected by N availability in the Wt, and about 80% of them are affected in an AreA and/or AreB-dependent manner (Fig 1A, S1 Table). Potential AreA/AreB binding sites (double GATA/TATC sequence elements with a distance of <30 bp) [23,38] were found in the promoters of 73% of all 14821 annotated *F*. *fujikuroi* genes, while 80% of the genes affected in the Δ*AREA* and/or Δ*AREB* mutant contained at least one pair of these motifs (S1 Table).

Under N-limiting conditions, 4,241 genes and 4,233 genes were regulated differentially in the Δ*AREA* and Δ*AREB* mutant, respectively, either being up- or down-regulated compared to the Wt (see S1 Table for details). Under N sufficiency, only six genes with currently unknown function exhibited differential regulation in the Δ*AREA* mutant, indicating that AreA does not play a significant role as a transcriptional regulator under these conditions. In contrast, a large set of 4,432 genes was affected in the Δ*AREB* mutant in the presence of sufficient N (S1 Table). 2,084 of these genes were affected in the Δ*AREB* mutant irrespective of N availability, suggesting an additional N-independent regulatory role of AreB.

Importantly, by using stricter selection criteria (> 4-fold change in expression at the 95% confidence interval), the deletion of *AREA* and *AREB* still affects the expression of large gene sets (S1f Table).

Analyzing the effects of AreA, AreB and nitrogen on individual gene sets using a two-way ANOVA, we found that nitrogen (P = 0.0031) and the interaction of AreA and nitrogen (P = 0.0149) have a significant effect on the expression of secondary metabolism genes [8]. In contrast, AreB significantly influences secondary metabolism gene expression (P = 0.0113) independent of nitrogen. We also found that nitrogen has a significant effect on secreted proteins (P = 0.0048) and that AreB affects gene expression of transcription factors (P = 0.0005).

In summary, our findings show that AreA and AreB are global regulators of large gene sets and that both may act either as activators or as repressors. The genome-wide transcriptome analysis also demonstrates that the function of AreB is much more complex than being a regulatory counterpart of AreA. Although the transcript level of *AREB* is significantly down-regulated under N-sufficient conditions (S1 Table) confirming our previous data [31], AreB but not AreA performs regulatory functions also under these conditions.

### Commonly affected genes in *ΔAREA* and *ΔAREB*: Elucidating AreA and AreB target genes

Previously we have shown that the expression of *AREB* depends on AreA, while AreB does not affect *AREA* expression [31], which was also reflected by our microarray analysis (S1 Table). This AreA-dependent transcriptional regulation of *AREB* makes it hard to distinguish between differentially expressed genes that are affected by AreA (referred to as ‘AreA targets’) and those that are affected by AreB (referred to as ‘AreB targets’). A gene that is affected in Δ*AREA* as well as in Δ*AREB* (Fig 1B) may be either a common target of both TFs or an AreB target only, which is indirectly affected in Δ*AREA* due to the drastically reduced *AREB* transcript levels in this mutant.

Therefore, we sorted the differentially expressed genes in the Δ*AREA* and Δ*AREB* mutants at N-limiting conditions into two major groups: (1) genes that are exclusively affected in one mutant and (2) genes that are affected in both mutants (Table 1, S2 Table). For the first group of genes, the differential expression is clearly the result of the lack of the respective TF, and the affected genes can be considered either as AreA or AreB targets, respectively. For the genes in the second group that are equally down- or up-regulated in both mutants, AreA might act only indirectly via down-regulation of *AREB*-expression (see Table 1 for details).

**Table 1:** Overview of all differentially expressed genes in Δ*AREA* and Δ*AREB* cultivated with 6 mM glutamine (N limitation).

gene set	No. of diff. regulated^a^ genes			Targets of?
general overview				
	**total**	**up**	**down**	
Genes affected in Δ*AREA*	**4241**	2192	2049	
Genes affected in Δ*AREB*	**4233**	2138	2095	
**(1)** affected in one mutant				
	**total**	**up**	**down**	
Genes affected in Δ*AREA* only	**2093**	1196	897	AreA
Genes affected in Δ*AREB* only	**2085**	1043	1042	AreB
**(2)** affected in both mutants				
	**total**	**up/down**	**down/up**	
antagonistic directed regulation in Δ*AREA*/Δ*AREB*	**357**	129	228	AreA, AreB
	**total**	**up/up**	**down/down**	
equally directed regulation in Δ*AREA*/Δ*AREB*	**1791**	867	924	AreB, AreA?

^a^ selection criteria: 2-fold change (up or down) in expression at the 95% confidence interval (False Discovery Rate < 0.05).

To shed light on the role each of the two TFs plays in regulating the expression of some of these unclear AreA target genes, we constitutively expressed *AREB* in the Δ*AREA* background (Δ*AREA*-*OE*::*AREB*), thereby circumventing the AreA-dependent expression of *AREB*. To verify functionality of the *OE*::*AREB* construct we complemented also the Δ*AREB* mutant with the same vector (Δ*AREB*-*OE*::*AREB*). As an example, we studied the expression of three genes which were strongly down-regulated in both deletion mutants: the key genes for GA (*CPS/KS*) and FUM (*FUM8*) biosynthesis, as well as the TF-encoding gene *FFUJ_04390*, a homologue of the *A*. *nidulans* nitrogen regulator gene *TAMA* [39,40] (Fig 1C). As expected, expression of the SM cluster genes was lost in Δ*AREA* as well as Δ*AREB* and restored in Δ*AREB*-*OE*::*AREB*. A similar expression pattern was observed for *TAMA*, which was significantly down-regulated but not completely lost in Δ*AREB*. Expression of none of these genes was restored in Δ*AREA*-*OE*::*AREB* indicating the essential role of both TFs for these genes. By this approach we provide for the first time experimental proof, that both AreA and AreB are indeed essential to synergistically activate expression of the GA and FUM gene clusters, going in line with our previous results where the expression of both SM gene clusters was strongly down-regulated in Δ*AREA* and Δ*AREB* mutants [13,20].

In conclusion, equally large sets of common but also distinct genes were affected in Δ*AREA* and Δ*AREB*. While TF-specific genes, and genes which were regulated by the two TFs in opposite directions, can clearly be assigned to the activity of one of the two TFs (AreA and/or AreB target genes), the impact of AreA on the equally regulated genes (Table 1) remains elusive. To dissect AreA function from that of AreB, the expression of a given gene needs to be studied in the Δ*AREA* mutant with constitutive, AreA-independent, expression of *AREB* as shown for the GA and FUM cluster genes and *TAMA* in this work.

### Impact of *AREA* and *AREB* deletion on the proteome

Apart from the transcriptional regulation, we examined the impact of AreA and AreB on protein abundance under both N conditions in biological duplicates, employing quantitative MS analysis and phosphoproteomics. Proteins were defined as significantly differentially expressed when the abundance of corresponding peptides was increased or decreased by log_2_FC 0.5, or when peptides were detected only in the Wt (down-regulated) or the mutant strains (up-regulated), respectively. The absence of peptides can be evaluated with high confidence due to advanced retention time alignment and enhancement as shown previously [41]. Overall, the strict selection criteria allow protein regulations to be identified with high biological significance.

In total 19,658 distinct peptides (posterior error probability (PEP) < = 1%) were identified, which correspond to 1,964 proteins encoded by the genome of *F*. *fujikuroi*. This set was subsequently refined to 1,010 proteins, for which at least two unique peptides were identified (S3 Table). Under N-limiting conditions, 446 proteins were differentially regulated in the Δ*AREA* mutant. Unexpectedly, a set of 138 proteins was affected in Δ*AREA* under N sufficiency, in contrast to the minor impact of AreA on gene expression under these conditions. In the Δ*AREB* mutant, 386 proteins were regulated differentially under N-limiting and 446 proteins under N-sufficient conditions (S1 Fig, Table 2, S3 Table). In a similar manner to the microarray analysis, we differentiated between proteins which are specifically up- or down-regulated only in one mutant (1), and others that are commonly affected in both (2) (Table 2).

**Table 2:** Overview of all differentially expressed proteins in Δ*AREA* and Δ*AREB* cultivated with 6 mM glutamine and 60 mM glutamine.

protein set	No. of diff. regulated^a^ proteins		Targets of?
general overview			
	**total 6**	**total 60**	
Proteins affected in Δ*AREA*	**446**	**138**	
Proteins affected in Δ*AREB*	**386**	**446**	
(1) exclusively affected in one mutant			
	**total 6**	**total 60**	
Proteins affected in Δ*AREA* only	**175**	**43**	AreA
Proteins affected in Δ*AREB* only	**115**	**351**	AreB
(2) commonly affected in both mutants			
	**total 6**	**total 60**	
antagonistic regulation in Δ*AREA* and Δ*AREB*	**38**	**16**	AreA, AreB
	**total 6**	**total 60**	
equally directed regulation in Δ*AREA* and Δ*AREB*	**233**	**79**	AreB, AreA?

^a^ selection criteria: 1-fold change (up or down) in protein abundance. Nitrogen-limiting and nitrogen-sufficient conditions are indicated by 6 (6 mM glutamine) and 60 (60 mM glutamine), respectively

Comparison between the proteome and transcriptome datasets revealed that a large set of proteins affected by N-availability or in the Δ*AREA* and/or Δ*AREB* mutants were not significantly regulated (log_2_FC < -1; > 1) on transcriptional level (S3 Table). In particular, more than 80% of the N-regulated proteins (Wt under N limitation compared with Wt under N sufficiency) were exclusively regulated at the protein level (S3 Table), suggesting the involvement of post-transcriptional regulations.

Taken together, the abundance of more than half of the quantified proteins was affected in the Δ*AREA* and/or the Δ*AREB* mutants, stressing the strong impact of both TFs on the proteome of *F*. *fujikuroi*. We also observed AreA- and AreB-mediated protein-regulations that were not affected at the transcriptional level, indicating the involvement of post-translational regulations.

### AreA and AreB regulate a large set of putative downstream regulators

To examine the hierarchical regulation networks, we were especially interested in downstream acting AreA and/or AreB-dependent TFs. From about 980 TFs identified in the genome of *F*. *fujikuroi* [8], 240 were differentially regulated in the Δ*AREA* mutant under N-limiting conditions (S4a Table). In the Δ*AREB* mutant, 311 and 296 TFs were affected under N-limiting and N-sufficient conditions, respectively, representing about 30% of the TFs in the genome under each condition (S4a Table). Examples of notable and strongly regulated TFs in Δ*AREA* and Δ*AREB* are shown in Table 3. Among them are TF-encoding genes which are putatively involved in N metabolism (e.g. TamA), C metabolism (e.g. homologs of Acu-15 involved in acetate metabolism [42]) as well as pathway-specific regulators belonging to certain SM-clusters (e.g. apicidin, beauvericin, bikaverin, DMATS1, fusaric acid, PKS1).

**Table 3:** Selection of differentially regulated transcription factor genes in Δ*AREA* and Δ*AREB* cultivated with 6 mM glutamine and 60 mM glutamine.

GENOME ID	CLASS	GATA PAIRS	ANNOTATION	FUNCTION/ SM-CLUSTER	WT 60 VS WT 6^a^	Δ*AREA* 6 VS WT 6^a^	Δ*AREB* 6 VS WT 6^a^	Δ*AREB* 60 VS WT 60^a^
FFUJ_00012	bZIP	2	Apf2—apicidin cluster transcription factor	apicidin cluster	9.48	N/A	2.07	-6.04
FFUJ_00054	Zn_2_Cys_6_	3	related to UPC2—regulatory protein involved in control of sterol uptake	sterol metabolism	N/A	4,82	2,37	N/A
FFUJ_02117	Zn_2_Cys_6_	4	Fub 10 –fusaric acid cluster transcription factor	fusaric acid cluster	9.99	1.67	2.30	-6.11
FFUJ_02119	Zn_2_Cys_6_	3	Fub 12 –fusaric acid cluster transcription factor	fusaric acid cluster	3.26	N/A	N/A	-2.22
FFUJ_02223	unknown	5	uncharacterized protein	PKS1 cluster	5.39	N/A	N/A	-3.60
FFUJ_02504	bZIP	2	related to SRP40—suppressor of mutant AC40 of RNA polymerase I and III	transcription/ splicing	N/A	4,23	3,15	3,42
FFUJ_02801	Cys_2_His_2_	1	related to krueppel protein Klp1	asexual development	N/A	-1.28	-1.78	-1.39
FFUJ_03660	Zn_2_Cys_6_	10	related to pathway-specific regulatory protein nit-4	nitrogen metabolism	-1,21	-2,79	-1,45	-3,62
FFUJ_04390	Zn_2_Cys_6_	10	TamA—transcriptional activator for allantoin and GABA catabolic genes	nitrogen metabolism	-2,36	-3,42	-3,29	-4,41
FFUJ_06522	Zn_2_Cys_6_	4	ARG81—Transcription factor involved in arginine metabolism	arginine metabolism	-4,55	-3,65	-1,19	N/A
FFUJ_06723	Zn_2_Cys_6_	0	related to transcriptional activator Mut3p	carbon metabolism	4,43	1,47	1,79	-4,30
FFUJ_08895	Zn_2_Cys_6_	2	related to transcriptional activator acu-15	carbon metabolism	N/A	-3,21	N/A	-1,40
FFUJ_09177	Zn_2_Cys_6_	0	uncharacterized transcription factor	DMATS1 cluster	-1.10	N/A	-1.40	N/A
FFUJ_09190	Zn_2_Cys_6_	4	related to STB5—SIN3 binding protein	carbon metabolism	-8,06	-8,19	1,09	N/A
FFUJ_09298	Zn_2_Cys_6_	19	Bea4—beauvericin cluster transcription factor	beauvericin cluster	-3.42	-1.75	N/A	N/A
FFUJ_11293	Cys_2_His_2_	0	related to TRI15—putative transcription factor	secondary metabolism	N/A	4,56	N/A	3,47
FFUJ_12023	Zn_2_Cys_6_	15	related to nitrate assimilation regulatory protein nirA	nitrogen metabolism	N/A	N/A	2,99	-6,71
FFUJ_12033	Zn_2_Cys_6_	4	related to transcription activator protein acu-15	carbon metabolism	N/A	N/A	-1,54	-3,49
FFUJ_12043	Zn_2_Cys_6_	1	related to transcriptional activator Mut3p	carbon metabolism	-1,55	N/A	-3,33	-3,39
FFUJ_12646	Zn_2_Cys_6_	3	related to transcriptional activator Mut3p	carbon metabolism	-1,67	-3,03	-3,04	-2,64
FFUJ_12938	Zn_2_Cys_6_	8	related to transcription activator protein acu-15	carbon metabolism	-3,01	-3,95	N/A	-2,03
FFUJ_12947	Zn_2_Cys_6_	6	related to C6 zink-finger protein PRO1A	sexual development	N/A	-2,48	-2,11	-4,25
FFUJ_13618	Zn_2_Cys_6_	0	related to thiamine repressible genes regulatory protein thi1	thiamine synthesis	1,45	1,36	3,34	1,60
FFUJ_13963	Zn_2_Cys_6_	3	Bik5—bikaverin cluster transcription factor	bikaverin cluster	-5,95	-3,22	1,55	1,60
FFUJ_14054	Zn_2_Cys_6_	2	related to transcription activator protein acu-15	carbon metabolism	N/A	N/A	N/A	-4,69
FFUJ_14818	Zn_2_Cys_6_	4	related to ARG81—transcription factor involved in arginine metabolism	arginine metabolism	-3,66	-3,39	-4,71	-2,69

^a^ RNA foldchange (FC) between two experimental conditions or strains in log2 scale (False Discovery Rate < 0.05). N/A indicates no significant foldchange (< 1, > -1) of gene expression under the respective condition. Nitrogen-limiting and nitrogen-sufficient conditions are indicated by 6 (6 mM glutamine) and 60 (60 mM glutamine), respectively.

Furthermore, we studied the impact of both GATA TFs on the expression of genes encoding potential histone-modifying enzymes (HMEs), such as histone methyltransferases and demethylases, as well as histone acetyltransferases (HAT) and deacetylases. Of 144 genes putatively encoding HME [8], about 35 and 20 were up- or down-regulated in the Δ*AREA* and Δ*AREB* mutant, respectively, under N-limiting conditions (S4b Table). Under N-sufficient conditions, AreB regulates 29 HME-encoding genes (S4b Table). Among the affected genes are *FFUJ_13544*, which is a homolog of SPT10, a yeast HAT [43] and the gene *FFUJ_08441*, which is a homolog to the major DNA methyltransferase Dim-2 of *N*. *crassa* [44].

The transcriptomics and proteomics data also indicated a regulatory function of both GATA TFs on putative protein kinases and phosphatases both on transcript and protein level (S1 and S3 Tables). We therefore searched for proteins that are phosphorylated in an AreA- and/or AreB-dependent manner by a phosphoproteomics approach. Among the 1,010 identified proteins, 277 were found to contain phosphorylated amino acids (S3 Table). However, we did not observe any differentially phosphorylated proteins in the Δ*AREA* and/or Δ*AREB* mutants compared to the Wt, indicating that AreA and AreB have no significant impact on the phosphorylation of the identified proteins.

These results suggest that both GATA TFs are on top of regulatory circuits, as they control the expression of a large set of genes encoding TFs and putative HMEs.

### AreA and AreB affect genes and proteins involved in several cellular processes

To gain a deeper insight into the most prominent functions of genes affected in Δ*AREA* and Δ*ARE*B, we performed a functional enrichment analysis using the MIPS ‘FunCat’ tool [45]. This analysis indicated an over-representation of genes likely involved in amino acid metabolism and disease, cellular transport, virulence and defense, and secondary metabolism (S5 Table). In contrast to AreA, AreB regulates many genes also at N sufficiency. This includes a significant number of genes involved in secondary metabolism, but most of the AreB target genes at high N are not yet characterized (S5 Table).

Similar to the transcriptome analysis, the majority of the proteins affected by the loss of AreA and/or AreB belong to the functional categories of secondary metabolism, but also amino acid and carbohydrate metabolism, including enzymes of the TCA and glyoxylate cycle (e.g. isocitrate-, malate-, and succinate dehydrogenases, isocitrate lyase) and the main enzymes of the glutamate/glutamine cycling (S2 Fig, S3 Table).

### AreA and AreB are major regulators of secondary metabolism

**Fig 2:**
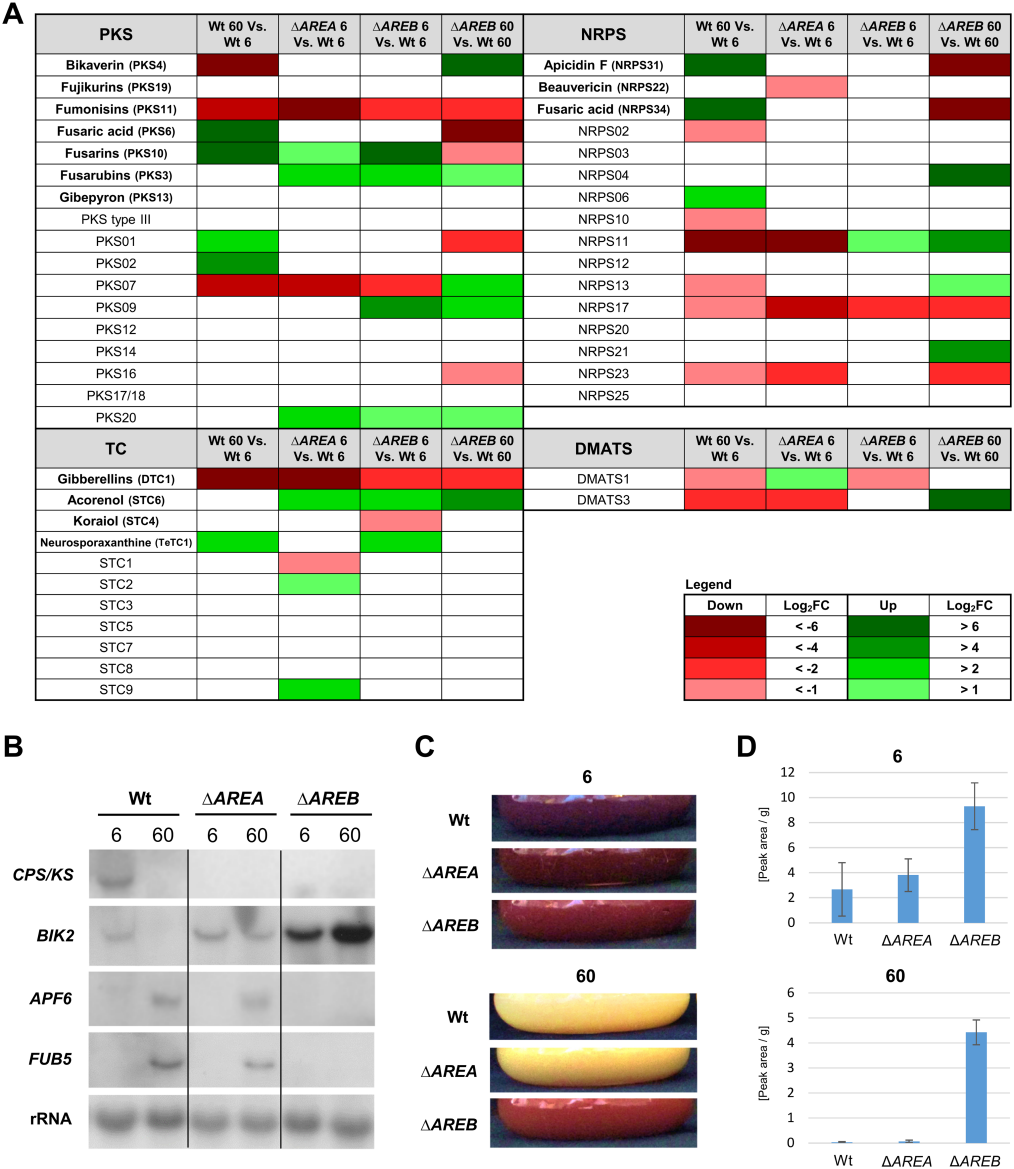
Influence of AreA and AreB on secondary metabolite cluster gene expression. The *F*. *fujikuroi* Wt and the Δ*AREA* and Δ*AREB* deletion mutants were cultivated for 3 (A, B, C) or 7 (D) days in ICI liquid cultures with 6 mM glutamine (6) or 60 mM glutamine (60) as sole nitrogen source. (A) Differentially regulated secondary metabolite gene clusters, represented by PKS-, NRPS-, TC- and DMATS-encoding genes. Data are based on microarray analysis. (B) Northern blot analysis of secondary metabolite cluster gene expression: *CPS/KS* (copalyl diphosphate/kaurene-synthase, GA-cluster), *BIK2* (monooxygenase, BIK-cluster), *APF6* (o-methyltransferase, APF-cluster), *FUB5* (homoserine o-acyltransferase, FUB-cluster). (C) Variation in pigmentation of the strains in ICI liquid culture. (D) Variation in bikaverin production. Culture supernatant was analyzed by HPLC-DAD. The peaks of bikaverin and norbikaverin were integrated at wavelength of 510 nm and depicted in relation to the total cell dry mass of the cultures.

The functional enrichment analysis revealed a major impact of both AreA and AreB on secondary metabolism in *F*. *fujikuroi*. Therefore, we studied the differential expression of the 47 predicted gene clusters under N-limiting as well as N-sufficient conditions in more detail (Fig 2A). The depicted expression of PKS-, TC-, NRPS- or DMATS-encoding genes is representative for the whole gene clusters. In the Wt, 12 SM gene clusters (BIK, FUM, PKS07, GA, NRPS02, NRPS10, NRPS11, NRPS13, NRPS17, NRPS23, DMATS1, DMATS3), were down-regulated, while the expression of eight SM gene clusters (FUB, FUS, PKS01, PKS02, neurosporaxanthine, APF, NRPS06) was elevated under N sufficiency. Of the 12 N-repressed SM clusters, seven were down-regulated in Δ*AREA* under inducing N-limiting conditions including the GA and FUM gene clusters. In addition, several cryptic gene clusters with yet unknown function are also significantly down-regulated in Δ*AREA*, e.g. PKS07, NRPS11, NRPS17, NRPS23 and DMATS3 (Fig 2A).

Compared to AreA, the impact of AreB on SM-regulation appears to be more diverse. Some of the N-repressed gene clusters, whose expression was significantly down-regulated in Δ*AREA*, e.g. GA, FUM, PKS07 and NRPS17, were also down-regulated in Δ*AREB*. For the GA and the FUM cluster the essential function of both GATA TFs regarding transcriptional activation has been demonstrated by constitutive expression of *AREB* in the Δ*AREA* background (Fig 1C), while the involvement of AreA in regulating PKS07 and NRPS17 still needs to be determined.

Interestingly, AreB appears to be essential also for the activation of the N-induced APF, PKS1 and FUB (PKS6; NRPS34) gene clusters, which were all down-regulated in Δ*AREB* (Fig 2A). In addition, AreB also acts as a transcriptional repressor of certain SM clusters. The PKS09, PKS20, α-acorenol and the neurosporaxanthine clusters, which are induced or unaffected by N in the Wt, are up-regulated under both conditions in Δ*AREB*. Furthermore, the N-repressed BIK, NRPS11 and DMATS3 clusters, were also significantly up-regulated in Δ*AREB* at N sufficiency, indicating a de-regulation of these SM under normally repressing conditions (Fig 2A).

To verify the microarray data, northern blot analyses under identical conditions were done using one of the co-regulated genes of the GA (*CPS/KS*), BIK (*BIK2*), APF (*APF6*) and FUB (*FUB5*) clusters as probes. The expression pattern of the tested clusters fully confirmed our findings from the microarray analysis: we recorded a complete loss of GA expression in both mutants, of APF and FUB gene expression in Δ*AREB* (Fig 2B), and an elevated expression of the BIK genes in the Δ*AREB* mutant under N sufficiency.

The most significant role of AreB as a repressor was observed for the bikaverin biosynthesis. Concomitant with elevated *BIK* transcription, the mycelium of the Δ*AREB* mutant was deeply red colored in liquid cultures under both N conditions (Fig 2C), indicating a de-regulation of BIK production under normally repressing N-sufficient conditions. HPLC-DAD analysis confirmed the expression data and revealed significantly elevated amounts of BIK under both N limitation and sufficiency in the Δ*AREB* mutant, while no significant differences have been observed between the Wt and the Δ*AREA* mutant (Fig 2D).

**Fig 3:**
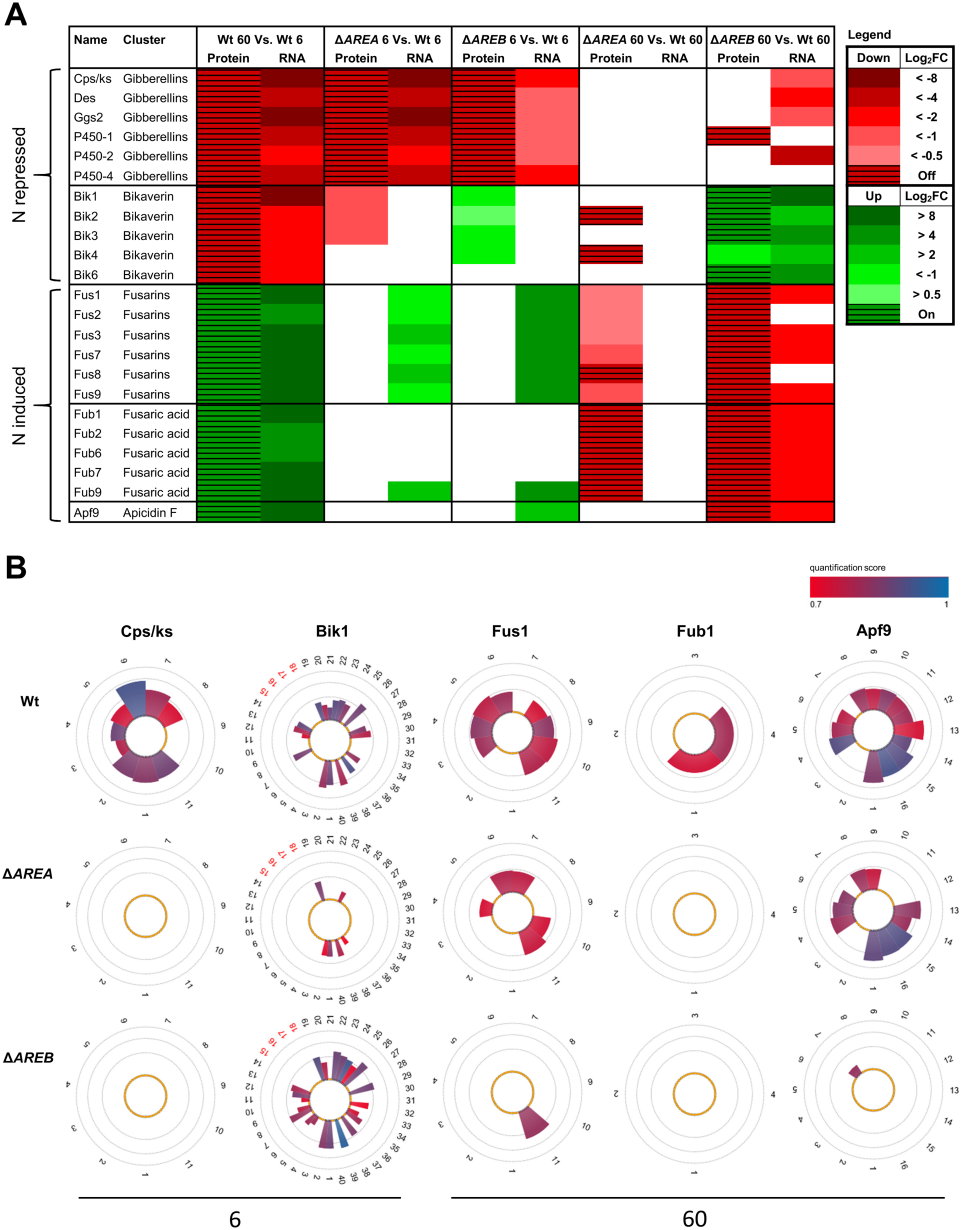
Comparison of AreA- and AreB-mediated regulation on secondary metabolite proteins and genes. The *F*. *fujikuroi* Wt and the Δ*AREA* and Δ*AREB* deletion mutants were cultivated for 3 days in ICI liquid cultures with 6 mM glutamine (6) or 60 mM glutamine (60) as sole nitrogen source. (A) Differentially regulated secondary metabolite proteins (Protein) and genes (RNA). Data is based on proteome and microarray analysis, respectively. (B) MS-based label free quantitation (pyQms) of peptides (numbers) detected during proteome analysis, corresponding to Cps/ks, Bik1, Fus1, Fub1 and Apf9 at 6 mM or 60 mM glutamine, respectively. A pie slice is used to represent each peptide; red numbering indicates phosphopeptides. The area of a pie slice correlates with the amount of the corresponding peptide, colors represent the quality of the pyQms quantification event. Results stemming from non-enriched samples of two independent experiments are depicted in log10 scale.

The observed different types of regulation of the GA, BIK, FUS, FUB and APF cluster genes in Δ*AREA* and Δ*AREB* correspond very well with the regulation of the respective proteins (Fig 3). These SM cluster genes are among the strongest regulated genes in both deletion mutants. High expression levels were shown to be a pre-condition for a good correlation between transcript and proteome data [46,47].

In conclusion, the transcriptomics and proteomics data demonstrate that AreA and AreB act as master regulators of secondary metabolism in *F*. *fujikuroi*. Both GATA TFs may function as transcriptional regulators of common, but also distinct, target SM clusters. While AreA acts exclusively as a strong positive regulator of N-repressed gene clusters (e.g. GA, FUM, PKS07 and NRPS17 genes) under N limitation, AreB can act as an activator (e.g. of APF, PKS1 and FUB genes) and repressor (e.g. of BIK, PKS09, NRPS04 and NRPS11 cluster genes) under both N conditions.

### Histone acetylation at some SM gene clusters is affected in Δ*AREA* and Δ*AREB*

Previously we have shown that the expression of several SM gene clusters in *F*. *fujikuroi* correlates with high levels of H3K9ac [6,8,9]. Histone acetylation is mediated by HAT complexes that are generally targeted to specific promoters through their physical interaction with sequence-specific TFs [48]. To study the possible role of AreA and AreB in the recruitment of HAT complexes in *F*. *fujikuroi*, we performed chromatin immunoprecipitation (ChIP) coupled to quantitative PCR using an anti-H3K9ac-specific antibody and measured the amount of precipitated DNA at four SM gene clusters that showed differential gene regulation in Δ*AREA* and/or Δ*AREB*. We chose the GA and FUM clusters (positively regulated by both TFs), FUB (positively regulated by AreB and only slightly affected in Δ*AREA*) and BIK (negatively regulated by AreB).

**Fig 4:**
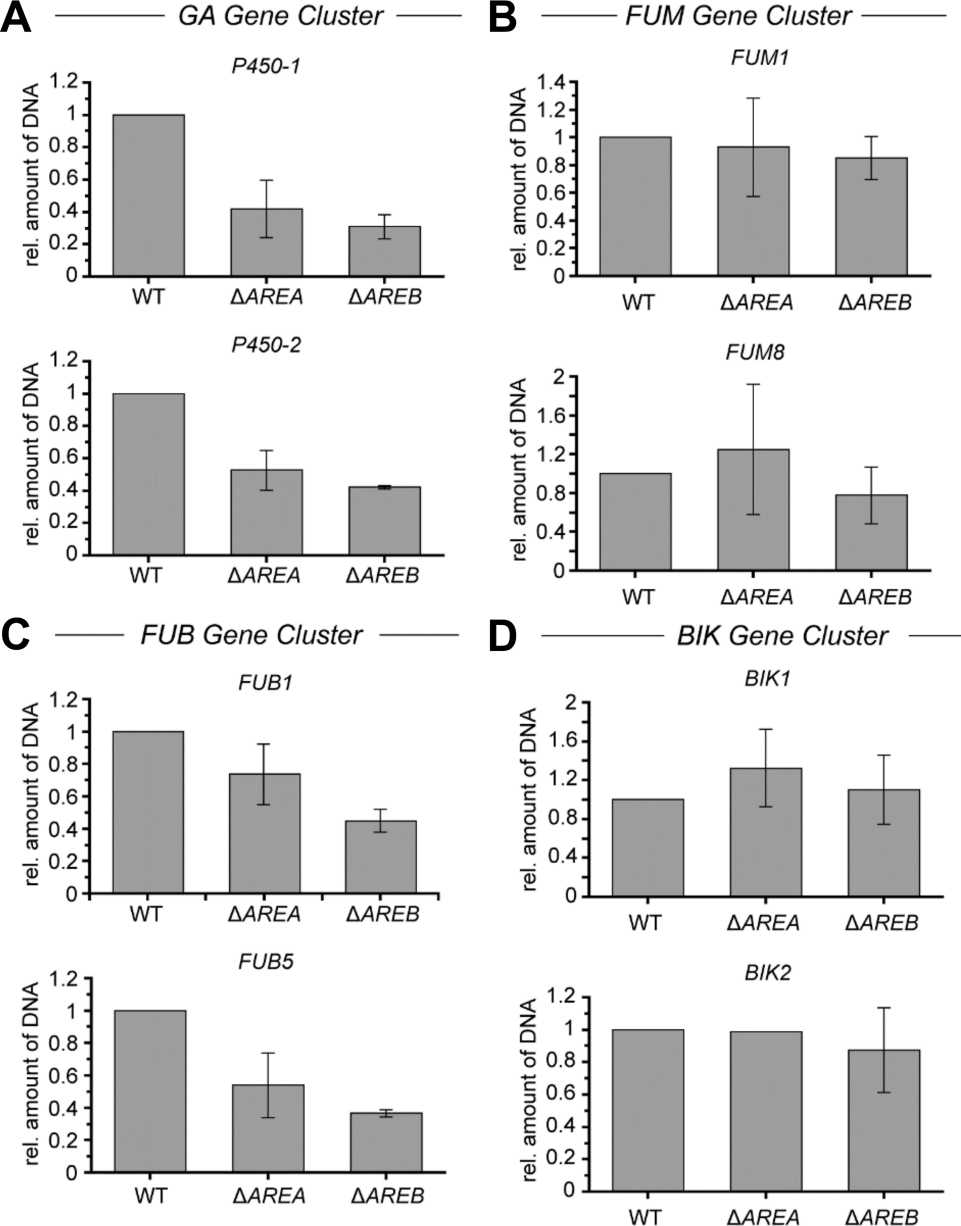
H3K9 acetylation is affected in Δ*AREA* and Δ*AREB* at the GA and FUB gene cluster. The *F*. *fujikuroi* Wt and the Δ*AREA* and Δ*AREB* deletion mutants were cultivated for 3 days in ICI liquid cultures with 6 mM (A, B, D) or 60 mM (C) glutamine as sole nitrogen source. The mycelium was subsequently cross-linked and used for chromatin immunoprecipitation (ChIP) experiments using an anti-H3K9ac antibody (AM39137). The precipitated amount of DNA was quantified at (A) GA (*P450-1* and *P450-2*), (B) FUM (*FUM1* and *FUM8*), (C) FA (*FUB1* and *FUB5*) and (D) BIK (*BIK1* and *BIK2*) cluster genes by qPCR. In each case, the amount of DNA in the Wt was arbitrarily set to 1. Mean values and standard deviations are shown. Experiments were done in technical and biological replicates.

As expected, the GA cluster genes *P450-1* and *P450-2* encoding P450 monooxygenases [49] showed significantly reduced H3K9ac levels upon deletion of *AREA* and *AREB*, suggesting that at least AreB is involved in HAT-mediated histone acetylation at the GA gene cluster (Fig 4A). Contrary to this finding, no significant differences in H3K9ac were observed at the FUM cluster genes *FUM1* and *FUM8* encoding the PKS [50] and an aminotransferase [51], respectively, albeit showing a similar reduced gene expression in Δ*AREA* and Δ*AREB* as the GA cluster genes (Fig 4B).

In a manner similar to GA cluster genes, H3K9ac levels were significantly reduced in the Δ*AREB* mutant also at the FUB genes *FUB1* and *FUB5* encoding the PKS and a putative homoserine-*O*-acyltransferase, respectively, in agreement with a significant decrease in FSA production [11]. Deletion of *AREA* led to only slightly reduced FSA biosynthesis and only slightly reduced H3K9ac levels at *FUB1* and *FUB5* (Fig 4C). H3K9ac levels remained almost unchanged at *BIK1* and *BIK5* upon deletion of *AREB* and *AREA*, under inducing (Fig 4D) as well as under repressing conditions (data not shown), albeit significantly enhanced BIK production in the *AREB*-deficient strain under both conditions, suggesting that additional *trans*-acting factors are involved in de-repression of BIK upon deletion of *AREB*.

Taken together, deletion of *AREA* and *AREB* results in reduced H3K9ac levels at GA and FUB cluster genes, which is in line with an abolished and reduced biosynthesis of GA and FUB, respectively. On the contrary, no correlation was observed between gene expression and H3K9ac levels in case of BIK and FUM clusters upon deletion of either *AREA* or *AREB*.

## Discussion

In this work, we studied the genome-wide regulatory impact of the two major N regulators, the GATA TFs AreA and AreB, in *F*. *fujikuroi* by quantitative transcriptomics and proteomics. By combining these techniques, we gained a deeper insight into the regulatory interplay of both TFs, their involvement in common but also different biological processes and their role in histone acetylation, which will be summarized and discussed in the following paragraphs.

### AreA and AreB are crucial regulators of nitrogen-dependent genes

AreA is considered as the major regulator of a large number of genes involved in uptake and metabolism of non-favored N sources in fungi [21,52,53]. Therefore, it is not surprising that AreA affects many genes and proteins that are involved in N metabolism and transport, including many putative N permeases, such as amino acid/peptide, ammonium, allantoin and GABA transporters. Our results show that most of these genes are activated by both GATA TFs under N-limiting conditions in *F*. *fujikuroi*. Much less is known about the function of the second N-responsive GATA TF, AreB. Recently, we have shown that AreB in *F*. *fujikuroi* is involved in regulating several processes of fungal life besides nitrogen metabolism, e.g. transport and secondary metabolism [31].

In this study, we provide the first comprehensive insight into the central role both TFs play in regulating metabolism using a genome-wide approach. About 80% of all N-regulated genes were affected by one or both TFs (Fig 1A). Interestingly, more than 4,000 genes and a large set of proteins were found to be up- or down-regulated by AreB at high N. In contrast to the gene expression data, we found that there is also a substantial number of AreA target proteins under these conditions (S3 Table, S1 and S2 Figs). This is an unexpected result because the expression of both GATA TF-encoding genes was shown to be repressed under N-sufficient conditions [31]. The most plausible explanation for this discrepancy might be that small quantities of AreA and AreB are probably still present under high N, which are sufficient to mediate the observed regulations. Furthermore, AreB regulates different sets of genes and proteins under N-limiting and N-sufficient conditions, and there are even examples of targets that are either activated or repressed by AreB in relation to the N availability. These different regulatory functions of AreB are most likely due to varying interaction partners and/or the three different transcript (*AREB-a*, *AREB-b*, *AREB-c*) and protein sizes which are formed depending on the N availability to the fungus [31]. While the shortest transcript (*AREB-c*) is the predominant form under N-limiting conditions, all three transcripts are present in equal proportions under N sufficiency. Furthermore, only the product of the largest transcript (AreB-a) has been detected inside the nucleus under N-sufficient conditions [31], indicating that the different regulatory functions of AreB at changing N conditions are probably be mediated by the products of the three different transcripts.

Surprisingly, AreA and AreB also regulate a large set of genes and proteins that are not affected by N availability, indicating that both GATA transcription factors play additional roles as regulators of N-independent processes. These data provide novel insights into additional roles of AreA besides being the major regulator of nitrogen metabolism and a master regulator of secondary metabolism in *F*. *fujikuroi*. Pleiotropic effects have also been described for AreA and AreB homologs in other filamentous fungi. In *A*. *nidulans*, AreB is involved in vegetative growth and asexual development [35] and plays a role in PacC-mediated pH regulation and virulence in *Colletotrichum gloeosporioides* [54]. In *Magnaporthe oryzae*, the GATA-type transcription factor Asd4 with high homology to AreB in *F*. *fujikuroi* is essential for sporulation, optimal growth on complete media and appressorium formation besides regulating genes involved in nitrogen assimilation [55]. AreA was shown to be essential for full virulence of the human pathogen *Aspergillus fumigatus* [56].

### Mode of action: Novel insights into the AreA-/AreB-dependent regulation

In *A*. *nidulans* and *P*. *chrysogenum* it was shown that AreA/NreA and AreB/NreB, can act as regulatory counterparts of shared target genes [32,35], which mimics the interplay of the activating AreA-orthologs Gln3p/Nil1p and the repressing AreB-orthologs Dal80p/Nil2p in yeast [57,58]. However, later it was exemplarily demonstrated in *A*. *nidulans* that both TFs act as synergistic repressors of arginine biosynthesis genes [36,37], and that AreB can be involved in the regulation of AreA-independent processes [35]. In this work, we have demonstrated for the first time on a genome-wide scale that the role of AreB is much more complex than simply being an antagonist of AreA: (1) AreB regulates a large set of specific target genes not affected by deletion of *AREA*, (2) AreB can act as positive as well as negative regulator, (3) the majority of genes and proteins affected in both mutants are regulated in the same way (either positively or negatively), (4) AreB, but not AreA, regulates a large set of genes also under N-sufficient conditions, (5) AreA and AreB have an impact on many genes whose expression is not affected by N availability.

For the genes and proteins that are activated or repressed by both TFs, it is hard to distinguish if alterations on transcript and protein levels are caused by the loss of AreA and/or AreB, or if they are indirectly affected by the down-regulation of *AREB* in the Δ*AREA* background. The expression of these potential targets should be further elucidated with the help of a constitutively expressed *AREB* in the Δ*AREA* deletion background, as we did for the GA and FUM cluster genes. These genes are among the most strongly down-regulated genes in Δ*AREA* and Δ*AREB*. Here we provide, for the first time, evidence that their expression clearly depends on the simultaneous presence of both GATA TFs, and that the strong impact of AreA is not due to the down-regulation of *AREB* in the Δ*AREA* mutant (Fig 1C). Recently, we have shown that AreA and AreB physically interact in the nucleus under N-limiting conditions. Such physical interaction is one possible explanation for the synergistic regulation of shared target genes and proteins [31]. We are currently investigating potential interaction partners of both GATA TFs by co-immunoprecipitation to better understand their mode of action.

### AreA- and AreB-mediated regulation of secondary metabolism

**Fig 5:**
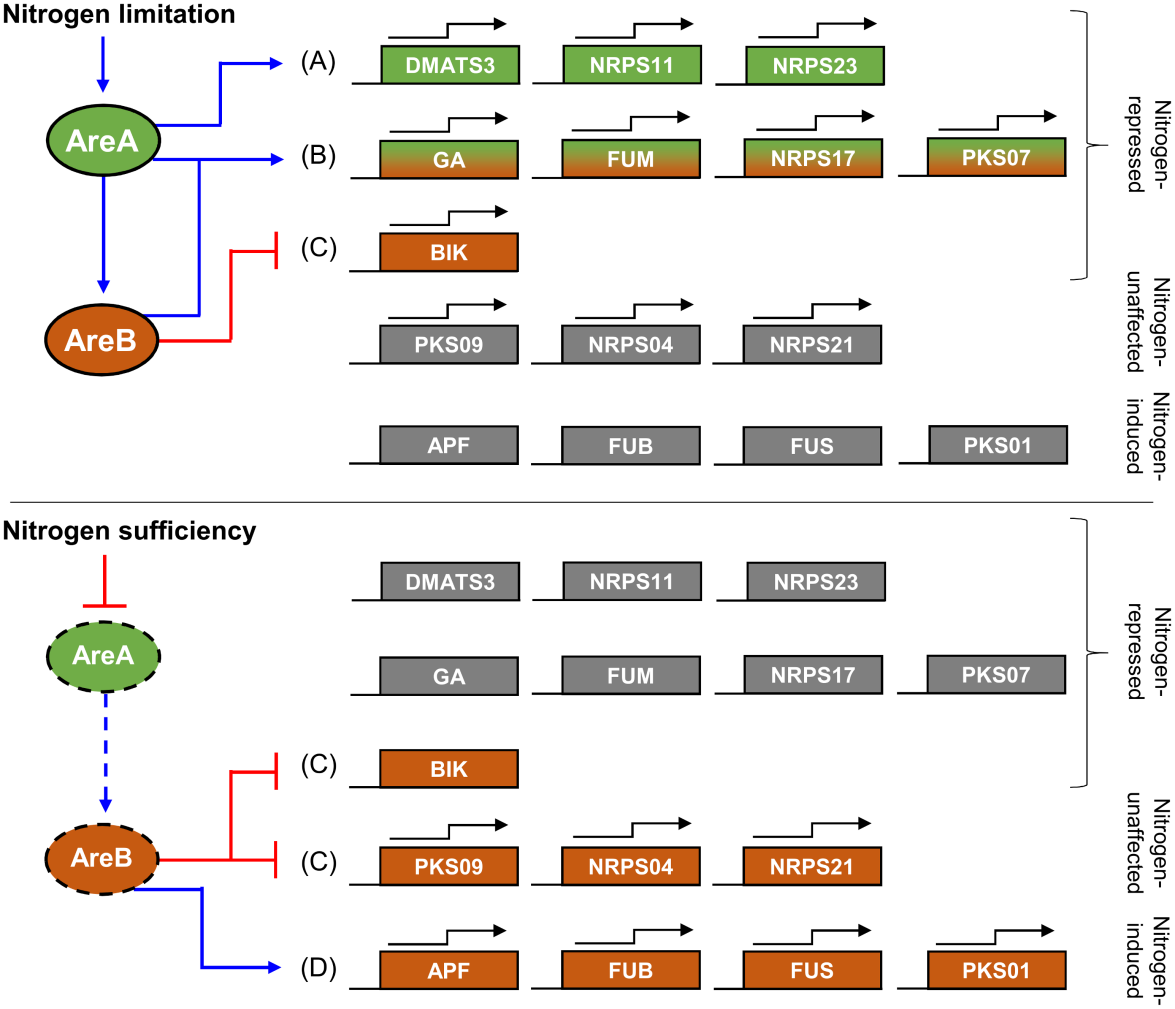
Overview of the main AreA- and AreB-mediated regulations of secondary metabolite cluster expression at nitrogen-limiting (6 mM glutamine) and nitrogen-sufficient (60 mM glutamine) conditions. Blue arrows indicate positive regulations, red lines indicate repressing effects. Black arrows indicate expression of clusters in the Wt under the respective nitrogen condition. (A) Clusters activated by AreA only; (B) clusters synergistically activated by AreA and AreB; (C) clusters repressed by AreB only; (D) clusters activated by AreB only.

Previously we have shown that AreA and/or AreB have an impact on the regulation of certain aspects of SM in *F*. *fujikuroi*, including GA, FSA, APF and FUM [10,11,13,20,31]. Here we show that AreA and/or AreB affect more than half of the genome’s SM biosynthetic pathways, which is summarized in Fig 5. While AreA mainly acts as a positive regulator of N-repressed clusters under N-limiting conditions (Fig 5A), the regulatory function of AreB appears to be more diverse because it regulates multiple genes independently of nitrogen availability as confirmed by two-way ANOVA analysis. AreB was shown to be a synergistic activator that is essential for expression of certain AreA target clusters at N-limiting conditions (Fig 5B). This was demonstrated for the GA and FUM clusters (Fig 1C) and is most likely true for the regulation of the NRPS17 cluster. However, AreB also regulates AreA-independent clusters and can act as a repressor (Fig 5C) as well as an activator (Fig 5D) under both N conditions. One of the most intriguing results is the high expression of BIK genes and proteins under otherwise repressing N-sufficient conditions in the Δ*AREB* mutant. Accordingly, AreB acts as a strong repressor of BIK biosynthesis, both under inducing low and repressing high N conditions. Besides BIK, AreB also plays a role as a repressor of the silent PKS09, NRPS04 and NRPS21 clusters (Fig 5C). Therefore, the Δ*AREB* deletion mutant could be a powerful tool to activate those gene clusters and to identify their products.

AreA and AreB could facilitate their regulatory impact on the SM-clusters by one of the following ways or combinations of them: (1) binding to the promoters of the single cluster genes, (2) binding to the promoter of a cluster-specific TF (if present) that activates expression of the remaining cluster genes and (3) activating or recruiting another global regulator that binds to the promoter of the cluster genes or mediates promoter accessibility by chromatin rearrangement. Previous work has indicated that AreB affects transcription of the APF [10] and FUS [11,14] clusters most likely via regulating the cluster-specific TF, which was confirmed by our microarray analysis (Table 3). Furthermore, we showed that AreB represses the TF-encoding gene *BIK5*, which indicates a similar regulation of the BIK cluster. For the GA cluster, which lacks a specific TF, it was experimentally shown that AreA directly binds to promoter sequences of the single cluster genes [20].

Besides direct binding to the promoters of target genes, AreB and possibly AreA also mediate histone acetylation at some regulated SM gene clusters. Acetylation of histones directs chromatin structural transitions from hetero- to euchromatin and thus directly affects the accessibility of the transcriptional machinery to the underlying DNA. There are several examples demonstrating a role of GATA-type TFs in the recruitment of HAT complexes. In mammalian systems, GATA-1 was shown to recruit histone H3 and H4 acetylation complexes during gene activation [59], and AreA has been linked to histone acetylation in *A*. *nidulans*. In the latter, H3K9 and H3K14 are acetylated at the nitrate gene cluster in a strictly AreA-dependent manner [28]. AreA also contributes to chromatin accessibility and expression of two *velvet*-regulated gene clusters, encoding biosynthesis of the mycotoxin beauvericin and of the siderophore ferricrocin in *F*. *oxysporum* [60]. However, a direct interaction between AreA and HAT complexes still awaits proof.

Here, our ChIP analysis revealed that the presence of AreB is required for acetylation of H3K9 at the GA gene cluster. A significant decrease in H3K9ac was also observed at the promoters of FUB genes. Reduction in H3K9ac was more pronounced in *AREB*- than in *AREA*-deficient strains, thereby mirroring production levels of both SM in both mutants. Therefore, AreB and possibly also AreA are required for proper H3K9ac and consequently GA and FUB gene expression in *F*. *fujikuroi*, in a similar manner to GATA-1 in mammals [59] and AreA in *A*. *nidulans* [28].

Contrary to GA and FUB genes, we detected no alteration in H3K9ac at the FUM cluster genes in Δ*AREA* and Δ*AREB* strains, albeit there was a complete loss of FUM biosynthesis [13]. However, recent data demonstrated that overexpression of the cluster-specific TF gene *FUM21* in the Δ*ARE*A and Δ*AREB* background leads to only partial restoration of FUM gene expression and supports our suggestion that AreA and AreB are involved in chromatin accessibility [13]. Similar to the *FUM* genes, no AreA/AreB-dependent changes in H3K9ac were observed at *BIK* genes, albeit higher gene expression in Δ*AREB*, suggesting that AreA and/or AreB do not facilitate H3K9ac at this SM gene cluster. Other histone modifications could be involved in the activation of the BIK and FUM clusters.

In conclusion, in addition to their role as major players mediating NMR, AreA and AreB can be considered as master regulators of secondary metabolism in *F*. *fujikuroi*. Both GATA factors are involved in the transcriptional regulation of more than half of the 47 SM clusters in the genome. While AreA mainly acts as a strong positive regulator of N-repressed clusters, the regulatory function of AreB appears to be more diverse, including repression of N-induced clusters. In addition, we could prove an involvement of AreB in H3K9 acetylation of some, but not all, AreA and/or AreB-regulated SM gene clusters.

## Materials and methods

### Fungal strains and culture conditions

In this study the following *F*. *fujikuroi* strains were used: wild-type (Wt) strain IMI58289 (Commonwealth Mycological Institute, Kew, UK), Δ*AREA*-T19 [100] and Δ*AREB*-T2.1 [39]. Strains were maintained on solid CM [61] and cultivated at 28°C in darkness. Submersive cultivation was performed as described in [62], with synthetic ICI (Imperial Chemical Industries, UK) minimal medium [63] supplemented with 6 mM or 60 mM glutamine as N source in biological duplicates. After cultivation for 72 h at 28°C, cultures were harvested and mycelium was used for extraction of total RNA or proteins and for ChIP analyses.

For yeast recombination cloning, *S*. *cerevisiae* strain FGSC9721/FY834 (*MATa his3Δ200 ura3-52 leu2Δ1 lys2Δ202 trplΔ63*) [64] was cultivated in 5 ml liquid YPD (pH 5.8, 10 g/l yeast extract, 20 g/l Bacto-Trypton (Difco), 20 g/l glucose) medium overnight at 200 rpm and 30°C. The culture was used to inoculate 50 ml liquid YPD and incubated at 200 rpm and 30°C for 4 to 6 h until an OD 600 nm of ~1 was reached. The harvested yeast cells were also used for yeast recombination cloning [65,66].

### Bacterial strains and plasmid construction

*Escherichia coli* strain Top10F’ (Invitrogen, Groningen, The Netherlands), cultivated in Lysogeny Broth medium [67] was used for plasmid propagation and amplification. For generating the pOE::AreB-GFP fusion vector, clones of the shortest *AREB* transcript of 1305 bp length (*AREB*-c [31]) were amplified using the primer AreB-GFP-F / AreB-GFP-R, which contain overlapping sequences homologous to the vector pNAH-OGG [66]. This vector contains a hygromycin resistance cassette, and a codon-optimized eGFP [68,69] under control of the constitutive *Aspergillus nidulans oliC* promoter and the *Botrytis cinerea gluc* terminator, respectively. The *AREB* PCR products and the NcoI-digested plasmid pNAH-OGG were co-transformed into *S*. *cerevisiae* yielding pOE::AreB-GFP by yeast recombination cloning (see above).

### Fungal transformations

Preparation of protoplasts of *F*. *fujikuroi* was carried out as described [70]. The Δ*AREA* and Δ*AREB* mutant strains were transformed with 20 μg of the pOE::AreB::GFP vector, yielding strains Δ*AREA-OE*::*AREB* and Δ*AREB-OE*::*AREB*. Transformed protoplasts were regenerated at 28°C in a complete regeneration agar (0.7 M sucrose, 0.5 g/l yeast extract) with 100 μg/ml nourseothricin (Werner Agents, Jena, Germany) or 100 μg/ml hygromycin (Sigma-Aldrich, Taufkirchen, Germany) for 4 to 7 days as specified above. Genomic integration of the overexpression-constructs was checked by diagnostic PCR with primers ogfp-seqR1 / AreB-seq3.

### Isolation of total RNA and northern blot

For northern blot analysis, total *F*. *fujikuroi* RNA was extracted from freeze-dried mycelium, using the RNAgents total RNA isolation kit (Promega, Mannheim, Germany). 20 μg of RNA were separated in a 1% agarose gel containing formaldehyde [67] and transferred to Hybond-N + membranes. Northern blot hybridizations were done by the method of Church and Gilbert [71]. For expression analysis of SM genes, corresponding probes were generated by PCR and ^32^P labeled using the random oligomer-primer method [67]. The following probes were used and amplified with the following primers: *APF6* (00008_aps6_F / 00008_aps6_R), *AREB* (areB-for-neu / areB-rev-neu), *BIK2* (bik2-F / bik2-R), *CPS/KS* (cps/ks-RT-for / cps/ks-RT-rev), *FUB5* (FF02109-Wt-F / FF02109-Wt-R), *FUM8* (fum8_F / fum8_R), *TAMA* (Tam1-F / Tam1-R).

### Microarray analysis

The *F*. *fujikuroi* microarray was designed by Roche NimbleGen Systems (Madison, WI) as described previously [8]. Microarray-hybridizations were performed at Arrows Biomedical (Münster, Germany) and RNA quality was checked using Agilent Bioanalyzer 2100 and RNA Nano 6000 Lab-Chip Lab-Chip Kit (Agilent Technologies).

Expression data were analyzed as described before [8]. Genes with an absolute log_2_-fold change above one or below minus one and an adjusted P-value (FDR) below 0.05 based on biological duplicates were regarded as significantly differentially expressed. The Munich Information Center for Protein Sequences Functional Catalogue (FunCat) [45] was used to identify biological processes. We applied Fisher’s exact test [72] to determine statistically overrepresented functional categories in differentially expressed gene sets. The retained P-values were adjusted using Bonferroni procedure. Tested categories with an adjusted P-value below 0.05 were regarded as significantly overrepresented in the gene set. The influence of nitrogen and genotype factor on gene sets was tested using a two-way Type-III ANOVA [73] at a significance level of P = 0.05. The analyzed gene sets were retrieved from [8].

### Analysis of bikaverin production by HPLC-DAD

Biological triplicates of strains were incubated for 7 days in ICI liquid cultures. Mycelium was harvested and culture supernatant was used for measurement of bikaverin and norbikaverin production by high-performance liquid chromatography coupled to a diode array detector (HPLC-DAD) at 510 nm wavelength. Samples were separated on a LiChrospher 100 RP-18 column (5 μm, 250 mm x 4 mm; Merck KGaA, Darmstadt, Germany) and measurement was performed on a Merck-Hitachi Chromatography System (Merck KGaA) fitted with an autosampler (L-7200), DAD (L-245) and gradient pump (L-7100). Chromatography conditions: solvent A ACN with 1% formic acid (v/v), solvent B 1% formic acid (v/v) with a flow rate of 1 ml/min. A gradient from 30% A to 45% A in 10 min, then for 15 min up to 50% A and followed by column flushing for 5 min at 100% A was used. Equilibration at the starting condition of 30% A was carried out for 3 min. Sample injection volume was 40 μl. Data were processed and analyzed using EZChrom Elite Version 3.3.2 SP1 (Scientific Software, Inc.)

### ChIP-coupled quantitative PCR

Mycelia of biological duplicates were crosslinked with 1% formaldehyde, incubated for 15 min at room temperature and 90 rpm, and quenched with 125 mM glycine. Mycelium was filtered over Miracloth, ground in liquid N and stored at -80°C until further use. ChIP was carried out with an anti H3K9ac-specific antibody (AM39137; Active Motif, CA 92008, United States) as described [74]. Precipitation of the protein-antibody conjugate was performed with Dynabeads^®^ Protein A (Novex^®^, Life Technologies). Precipitated DNA was quantified by qPCR according to protocol (Bio-Rad) using iQ^™^ SYBR^®^ Green Supermix (Bio-Rad) and normalized to input DNA. Primers used for qPCR are listed in S6 Table. Primer efficiencies were kept between 90 and 110% and experiments were done in technical and biological replicates. Sequences of all *F*. *fujikuroi* ORFs were extracted from the publicly available genome sequence of *F*. *fujikuroi* [8].

### Proteome analysis

Total protein was extracted from freeze-dried mycelium as described before [75]. 200 μg of total protein from the *F*. *fujikuroi* Wt, the Δ*AREA* and Δ*AREB* mutants (biological duplicates) were digested with trypsin according to the FASP method [76] followed by TiO_2_ phosphopeptide enrichment as already described [77].

#### LC-MS/MS measurement

Chromatographic separation of peptides was performed using an Ultimate 3000 RSLCnano System (Dionex, part of Thermo Fisher Scientific). The mobile phases for the loading pump consisted of 0.05% (v/v) TFA in ultrapure water (A) and 80% ACN/0.05% TFA in ultrapure water (B). The sample (1 μl) was loaded on a trapping column (C18 PepMap 100, 300 μM x 5 mm, 5 μm particle size, 100 Å pore size; Thermo Scientific) and desalted for 5 min using eluent A at a flow rate of 20 μl/min. Then the trap column was switched online with the separation column (Acclaim PepMap100 C18, 75 μm x 15 cm, 2 μM particle size, 100 Å pore size, Thermo Scientific). The mobile phases for elution of the peptides from the column consisted of 0.1% (v/v) formic acid in ultrapure water (A*) and 80% ACN/0.08% formic acid in ultrapure water (B*). Peptides were eluted at a flow rate of 300 nl/min and employing the following gradient profile: 0–40% B* over 90 min, 40–100% B* over 5 min, 100% B* for 10 min. Afterwards the column was re-equilibrated with 99% A* for 20 min.

The LC system was coupled via a nanospray source to an LTQ Orbitrap XL mass spectrometer (Thermo Finnigan). The general mass spectrometric conditions were: spray voltage, 1.5 kV; no sheath and auxiliary gas flow; ion transfer tube temperature: 200°C. The mass spectrometer was operated in positive ion mode and a data-dependent automatic switch was employed between MS and MS/MS acquisition modes. MS full scans (m/z 375–1600) were acquired in positive ion mode by FT-MS in the Orbitrap at a resolution of 60,000 (FWHM) with internal lock mass calibration on m/z 445.12003. The 12 most intense ions were fragmented in the linear ion trap by CID (35% normalized collision energy).

For standard samples, automatic gain control (AGC) was enabled with target values of 5 x 105 and 5 x 104 for MS full scans and MS/MS, respectively. One microscan was acquired per MS/MS spectrum and maximum ion trap fill time was 100 ms [78]. Ion selection thresholds were: 500 counts for MS2. An activation q = 0.25 and activation time of 30 ms were applied in MS2 acquisitions. Dynamic exclusion was enabled with an exclusion duration of 90s, repeat count of 1, list size of 500 and exclusion mass width of +/- 5 ppm. Unassigned charge states and charged state 1 were rejected.

To improve the fragmentation of phosphopeptides, multi-stage activation (MSA) in the Xcalibur software was enabled for each MS/MS spectrum. The 5 most intense ions were fragmented in the linear ion trap by CID (35% normalized collision energy). For the Multi Stage Activation (MSA), an MSA for further fragmention of the ions was triggered if in the 5 most intense peaks of a MS2 event a neutral loss peak at -98, -49 or -32.7 Da was observed. Automatic gain control (AGC) was enabled with target values of 5 x 105 and 5 x 104 for MS full scans and MS/MS, respectively. Two microscans were acquired per MS/MS spectrum and maximum ion trap fill time was 150 ms. Ion selection thresholds were: 500 counts for MS2. An activation q = 0.25 and activation time of 30 ms were applied in MS2 acquisitions. Dynamic exclusion was enabled with an exclusion duration of 120 s, repeat count of 1, list size of 500 and exclusion mass width of +/- 5 ppm. Unassigned charge states and charged state 1 were rejected. Additionally, Phosphopeptide-enriched samples (FT, E1 and E2) were measured with the following adjustments: Automatic gain control (AGC) was enabled with target values of 5 x 105 and 5 x 104 for MS full scans and MS/MS, respectively. Two microscans were acquired per MS/MS spectrum and maximum ion trap fill time was 150 ms.

#### Data analysis

Data analysis was performed as described previously [77]. LC-MS/MS data was converted from RAW format (Thermo scientific) to the open mzML format [79,80] using msconvert (Proteowizard version 2.0.1885 [81]). Then, mzML files were converted to the mascot generic format (mgf) if required using pymzML [82]. Peptide spectrum matches (PSMs) and statistical post processing was performed using Ursgal [83]. The following peptide database search algorithms were employed: OMSSA (version 2.1.9) [84], X! Tandem (version sledgehammer) [85], and MS-GF+ (version 9979 [86] using default values for most parameters (see http://ursgal.readthedocs.io/). A shuffled-peptide based target-decoy database was generated as described previously [87] based on *Fusarium fujikuroi* database (p3_i2_t5127_Fus_fujik_v21.prot version 21, [8]) and the contaminant database (cRAP, http://www.thegpm.org/crap/). Variable modifications were set as follows: oxidation of methionine (+15.9949 Da), acetylation of the N-terminus (+42.0106 Da), phosphorylation of serine, tyrosine and threonine (+79.966331). Additionally, X! Tandem considers the loss of water (-18.0106 Da) and deamidation (-17.0265 Da) by default. Carbamidomethylation of cysteine was set as fixed modification. Two missed cleavage sites were permitted. Statistical post-processing and the estimation of posterior error probabilities (PEP) for individual PSMs was performed using Percolator [88,89]. All PSMs were filtered on database search engine level with a cutoff ≤ 1% PEP. A total of 19658 unique peptides were identified (PEP ≤ 1%). Quantification was performed using pyQms with default parameters [87,90]. Retention time was aligned as described previously [87,90]. Intensity alignment and ratio calculation was performed as described previously [65]. Illustrations of logical relations between sets of regulated genes and proteins (Venn diagrams) were created using Ursgal [83]. Linear fits and coefficients of determination between gene ratios stemming from microarray data and protein ratios stemming from proteomics data was calculated using the linregress function of Scipy (https://www.scipy.org/).

## Supporting information

S1 Table Complete list of differentially regulated genes in the *F*. *fujikuroi* Wt, the Δ*AREA* and Δ*AREB* deletion mutants under N-limiting (6 mM gln) and N-sufficient (60 mM) conditions. (XLSX)

S2 Table Lists of differentially expressed genes under N-limiting (6 mM gln) conditions that are either affected exclusive in Δ*AREA*, Δ*AREB* or in both mutants. (XLSX)

S3 Table Complete list of differentially regulated proteins in the *F*. *fujikuroi* Wt, the Δ*AREA* and Δ*AREB* deletion mutants under N-limiting (6 mM glutamine) and N-sufficient (60 mM glutamine) conditions. (XLSX)

S4 Table Differentially regulated genes encoding predicted transcription factors (S4a) and histone modifying enzymes (S4b) in the *F*. *fujikuroi* Wt, the Δ*AREA* and Δ*AREB* deletion mutants under N-limiting (6 mM gln) and N-sufficient (60 mM) conditions. (XLSX)

S5 Table ‘Fun Cat’ analysis of differentially regulated genes in the *F*. *fujikuroi* Wt, the Δ*AREA* and Δ*AREB* deletion mutants under N-limiting (6 mM glutamine) and N-sufficient (60 mM glutamine) conditions. (XLSX)

S6 Table List of primers used in this study. (DOCX)

S1 Fig Venn-diagrams representing the distribution of proteins affected in Δ*AREA* and Δ*AREB*, as well as by nitrogen availability. The *F*. *fujikuroi* Wt and the Δ*AREA* and Δ*AREB* deletion mutants were cultivated for 3 days in ICI liquid cultures with 6 mM (Nitrogen limitation) or 60 mM (nitrogen sufficiency) glutamine as sole nitrogen source. Data is based on proteome analysis. Shown are differentially up-regulated and down-regulated proteins at nitrogen limitation (6) and nitrogen sufficiency (60) in Δ*AREA* and Δ*AREB* compared to Wt.
(TIF)

S2 Fig Functional category distribution of proteins affected in Δ*AREA* and Δ*AREB*. The *F*. *fujikuroi* Wt and the Δ*AREA* and Δ*AREB* deletion mutants were cultivated for 3 days in ICI liquid cultures with 6 mM (-N) or 60 mM (+N) gln as sole nitrogen source. Data is based on proteome analysis. Differentially regulated proteins in the mutants compared to Wt under respective nitrogen conditions were functionally classified according to their most prominent FunCat category. Stippled areas represent proteins affected in Δ*AREA*, which are regulated on an equal level (+/- 20%) or stronger in Δ*AREB*. For these proteins, the regulations might be an indirect effect due to the reduced levels of AreB in the Δ*AREA* mutant.
(TIF)

## References

[1] Sun SK, Snyder WC. The bakanae disease of the rice plant Fusarium: diseases, biology and taxonomy The Pennsylvania State University Press, University Park 1981; 104–113.

[2] Kurosawa E. Experimental studies on the nature of the substance secreted by the bakanae fungus. Nat Hist Soc Formosa. 1926;16: 213–227.

[3] Yabuta T. Biochemistry of the “bakanae” fungus of rice. Agric Hortic. 1935;10: 17–22.

[4] Tudzynski B, Hölter K. Gibberellin biosynthetic pathway in *Gibberella fujikuroi*: evidence for a gene cluster. Fungal Genet Biol. 1998;25: 157–170. 10.1006/fgbi.1998.1095 9917370

[5] Wiemann P, Willmann A, Straeten M, Kleigrewe K, Beyer M, Humpf H-U, et al Biosynthesis of the red pigment bikaverin in *Fusarium fujikuroi*: genes, their function and regulation. Mol Microbiol. 2009;72: 931–946. 10.1111/j.1365-2958.2009.06695.x 19400779

[6] Studt L, Wiemann P, Kleigrewe K, Humpf H-U, Tudzynski B. Biosynthesis of fusarubins accounts for pigmentation of *Fusarium fujikuroi* perithecia. Appl Environ Microbiol. 2012;78: 4468–4480. 10.1128/AEM.00823-12 22492438 PMC3370568

[7] Desjardins AE, Plattner RD, Nelson PE. Production of fumonisin B and moniliformin by *Gibberella fujikuroi* from rice from various geographic areas. Appl Environ Microbiol. 1997;63: 1838–1842. 16535599 10.1128/aem.63.5.1838-1842.1997 PMC1389154

[8] Wiemann P, Sieber CMK, von Bargen KW, Studt L, Niehaus E-M, Espino JJ, et al Deciphering the cryptic genome: genome-wide analyses of the rice pathogen *Fusarium fujikuroi* reveal complex regulation of secondary metabolism and novel metabolites. PLoS Pathog. 2013;9: e1003475 10.1371/journal.ppat.1003475 23825955 PMC3694855

[9] Niehaus E-M, Kleigrewe K, Wiemann P, Studt L, Sieber CMK, Connolly LR, et al Genetic manipulation of the *Fusarium fujikuroi* fusarin gene cluster yields insight into the complex regulation and fusarin biosynthetic pathway. Chem Biol. 2013;20: 1055–1066. 10.1016/j.chembiol.2013.07.004 23932525

[10] Niehaus E-M, Janevska S, von Bargen KW, Sieber CMK, Harrer H, Humpf H-U, et al Apicidin F: characterization and genetic manipulation of a new secondary metabolite gene cluster in the rice pathogen *Fusarium fujikuroi*. PLoS ONE. 2014;9: e103336 10.1371/journal.pone.0103336 25058475 PMC4109984

[11] Niehaus E-M, von Bargen KW, Espino JJ, Pfannmüller A, Humpf H-U, Tudzynski B. Characterization of the fusaric acid gene cluster in *Fusarium fujikuroi*. Appl Microbiol Biotechnol. 2014;98: 1749–1762. 10.1007/s00253-013-5453-1 24389666

[12] Nazari F, Sulyok M, Kobarfard F, Yazdanpanah H, Krska R. Evaluation of emerging *Fusarium* mycotoxins beauvericin, enniatins, fusaproliferin and moniliformin in domestic rice in Iran. Iran J Pharm Res. 2015;14: 505–512. 25901158 PMC4403067

[13] Rösler SM, Sieber CMK, Humpf H-U, Tudzynski B. Interplay between pathway-specific and global regulation of the fumonisin gene cluster in the rice pathogen *Fusarium fujikuroi*. Appl Microbiol Biotechnol. 2016;100: 5869–82. 10.1007/s00253-016-7426-7 26966024

[14] Studt L, Janevska S, Niehaus E-M, Burkhardt I, Arndt B, Sieber CMK, et al Two separate key enzymes and two pathway-specific transcription factors are involved in fusaric acid biosynthesis in *Fusarium fujikuroi*. Environ Microbiol. 2016;18: 936–956. 10.1111/1462-2920.13150 26662839

[15] Niehaus E-M, Studt L, von Bargen KW, Kummer W, Humpf H-U, Reuter G, et al Sound of silence: the beauvericin cluster in Fusarium fujikuroi is controlled by cluster-specific and global regulators mediated by H3K27 modification. Environ Microbiol. 2016;18: 4282–4302. 10.1111/1462-2920.13576 27750383

[16] Haas H, Marzluf GA. NRE, the major nitrogen regulatory protein of *Penicillium chrysogenum*, binds specifically to elements in the intergenic promoter regions of nitrate assimilation and penicillin biosynthetic gene clusters. Curr Genet. 1995;28: 177–183. 8590470 10.1007/BF00315785

[17] Min K, Shin Y, Son H, Lee J, Kim J-C, Choi GJ, et al Functional analyses of the nitrogen regulatory gene *areA* in *Gibberella zeae*. FEMS Microbiol Lett. 2012;334: 66–73. 10.1111/j.1574-6968.2012.02620.x 22702217

[18] Giese H, Sondergaard TE, Sørensen JL. The AreA transcription factor in *Fusarium graminearum* regulates the use of some nonpreferred nitrogen sources and secondary metabolite production. Fungal Biol. 2013;117: 814–821. 10.1016/j.funbio.2013.10.006 24295920

[19] Li J, Pan Y, Liu G. Disruption of the nitrogen regulatory gene *AcareA* in *Acremonium chrysogenum* leads to reduction of cephalosporin production and repression of nitrogen metabolism. Fungal Genetics and Biology. 2013;61: 69–79. 10.1016/j.fgb.2013.10.006 24161729

[20] Mihlan M, Homann V, Liu T-WD, Tudzynski B. AREA directly mediates nitrogen regulation of gibberellin biosynthesis in *Gibberella fujikuroi*, but its activity is not affected by NMR. Mol Microbiol. 2003;47: 975–991. 12581353 10.1046/j.1365-2958.2003.03326.x

[21] Kudla B, Caddick MX, Langdon T, Martinez-Rossi NM, Bennett CF, Sibley S, et al The regulatory gene *areA* mediating nitrogen metabolite repression in *Aspergillus nidulans*. Mutations affecting specificity of gene activation alter a loop residue of a putative zinc finger. EMBO J. 1990;9: 1355–1364. 1970293 10.1002/j.1460-2075.1990.tb08250.x PMC551819

[22] Fu YH, Marzluf GA. Site-directed mutagenesis of the “zinc finger” DNA-binding domain of the nitrogen-regulatory protein NIT2 of *Neurospora*. Mol Microbiol. 1990;4: 1847–1852. 2150539 10.1111/j.1365-2958.1990.tb02033.x

[23] Ravagnani A, Gorfinkiel L, Langdon T, Diallinas G, Adjadj E, Demais S, et al Subtle hydrophobic interactions between the seventh residue of the zinc finger loop and the first base of an HGATAR sequence determine promoter-specific recognition by the *Aspergillus nidulans* GATA factor AreA. EMBO J. 1997;16: 3974–3986. 10.1093/emboj/16.13.3974 9233807 PMC1170021

[24] Caddick MX, Arst HN. Deletion of the 389 N-terminal residues of the transcriptional activator AREA does not result in nitrogen metabolite derepression in *Aspergillus nidulans*. J Bacteriol. 1998;180: 5762–5764. 9791130 10.1128/jb.180.21.5762-5764.1998 PMC107639

[25] Tudzynski B. Nitrogen regulation of fungal secondary metabolism in fungi. Front Microbiol. 2014;5: 656 10.3389/fmicb.2014.00656 25506342 PMC4246892

[26] Narendja F, Goller SP, Wolschek M, Strauss J. Nitrate and the GATA factor AreA are necessary for in vivo binding of NirA, the pathway-specific transcriptional activator of *Aspergillus nidulans*. Mol Microbiol. 2002;44: 573–583. 11972792 10.1046/j.1365-2958.2002.02911.x

[27] Muro-Pastor MI, Strauss J, Ramón A, Scazzocchio C. A paradoxical mutant GATA factor. Eukaryotic Cell. 2004;3: 393–405. 10.1128/EC.3.2.393-405.2004 15075269 PMC387643

[28] Berger H, Basheer A, Böck S, Reyes-Dominguez Y, Dalik T, Altmann F, et al Dissecting individual steps of nitrogen transcription factor cooperation in the *Aspergillus nidulans* nitrate cluster. Mol Microbiol. 2008;69: 1385–1398. 10.1111/j.1365-2958.2008.06359.x 18673441

[29] Muro-Pastor MI, Gonzalez R, Strauss J, Narendja F, Scazzocchio C. The GATA factor AreA is essential for chromatin remodelling in a eukaryotic bidirectional promoter. EMBO J. 1999;18: 1584–1597. 10.1093/emboj/18.6.1584 10075929 PMC1171246

[30] Berger H, Pachlinger R, Morozov I, Goller S, Narendja F, Caddick M, et al The GATA factor AreA regulates localization and in vivo binding site occupancy of the nitrate activator NirA. Mol Microbiol. 2006;59: 433–446. 10.1111/j.1365-2958.2005.04957.x 16390440

[31] Michielse CB, Pfannmüller A, Macios M, Rengers P, Dzikowska A, Tudzynski B. The interplay between the GATA transcription factors AreA, the global nitrogen regulator and AreB in *Fusarium fujikuroi*. Mol Microbiol. 2014;91: 472–493. 10.1111/mmi.12472 24286256

[32] Haas H, Angermayr K, Zadra I, Stöffler G. Overexpression of *nreB*, a new GATA factor-encoding gene of *Penicillium chrysogenum*, leads to repression of the nitrate assimilatory gene cluster. J Biol Chem. 1997;272: 22576–22582. 9278412 10.1074/jbc.272.36.22576

[33] Conlon H, Zadra I, Haas H, Arst HN, Jones MG, Caddick MX. The *Aspergillus nidulans* GATA transcription factor gene *areB* encodes at least three proteins and features three classes of mutation. Mol Microbiol. 2001;40: 361–375. 11309119 10.1046/j.1365-2958.2001.02399.x

[34] Wong KH, Hynes MJ, Davis MA. Recent advances in nitrogen regulation: a comparison between *Saccharomyces cerevisiae* and filamentous fungi. Eukaryot Cell. 2008;7: 917–925. 10.1128/EC.00076-08 18441120 PMC2446658

[35] Wong KH, Hynes MJ, Todd RB, Davis MA. Deletion and overexpression of the *Aspergillus nidulans* GATA factor AreB reveals unexpected pleiotropy. Microbiology (Reading, Engl). 2009;155: 3868–3880. 10.1099/mic.0.031252-0 19628561

[36] Dzikowska A, Kacprzak M, Tomecki R, Koper M, Scazzocchio C, Weglenski P. Specific induction and carbon/nitrogen repression of arginine catabolism gene of *Aspergillus nidulans*—functional in vivo analysis of the *otaA* promoter. Fungal Genet Biol. 2003;38: 175–186. 12620254 10.1016/s1087-1845(02)00522-4

[37] Macios M, Caddick MX, Weglenski P, Scazzocchio C, Dzikowska A. The GATA factors AREA and AREB together with the co-repressor NMRA, negatively regulate arginine catabolism in *Aspergillus nidulans* in response to nitrogen and carbon source. Fungal Genet Biol. 2012;49: 189–198. 10.1016/j.fgb.2012.01.004 22300944

[38] Merika M, Orkin SH. DNA-binding specificity of GATA family transcription factors. Mol Cell Biol. 1993;13: 3999–4010. 8321207 10.1128/mcb.13.7.3999-4010.1993 PMC359949

[39] Davis MA, Small AJ, Kourambas S, Hynes MJ. The *tamA* gene of *Aspergillus nidulans* contains a putative zinc cluster motif which is not required for gene function. J Bacteriol. 1996;178: 3406–3409. 8655534 10.1128/jb.178.11.3406-3409.1996 PMC178106

[40] Small AJ, Todd RB, Zanker MC, Delimitrou S, Hynes MJ, Davis MA. Functional analysis of TamA, a coactivator of nitrogen-regulated gene expression in *Aspergillus nidulans*. Mol Genet Genomics. 2001;265: 636–646. 11459183 10.1007/s004380100456

[41] Kukuczka B, Magneschi L, Petroutsos D, Steinbeck J, Bald T, Powikrowska M, et al Proton Gradient Regulation5-like1-mediated cyclic electron flow is crucial for acclimation to anoxia and complementary to nonphotochemical quenching in stress adaptation. Plant Physiol. 2014;165: 1604–1617. 10.1104/pp.114.240648 24948831 PMC4119042

[42] Bibbins M, Crepin VF, Cummings NJ, Mizote T, Baker K, Mellits KH, et al A regulator gene for acetate utilisation from Neurospora crassa. Mol Genet Genomics. 2002;267: 498–505. 10.1007/s00438-002-0682-5 12111557

[43] Kurat CF, Lambert J-P, Petschnigg J, Friesen H, Pawson T, Rosebrock A, et al Cell cycle-regulated oscillator coordinates core histone gene transcription through histone acetylation. Proc Natl Acad Sci USA. 2014;111: 14124–14129. 10.1073/pnas.1414024111 25228766 PMC4191790

[44] Kouzminova E, Selker EU. *dim-2* encodes a DNA methyltransferase responsible for all known cytosine methylation in *Neurospora*. EMBO J. 2001;20: 4309–4323. 10.1093/emboj/20.15.4309 11483533 PMC149169

[45] Ruepp A, Zollner A, Maier D, Albermann K, Hani J, Mokrejs M, et al The FunCat, a functional annotation scheme for systematic classification of proteins from whole genomes. Nucl Acids Res. 2004;32: 5539–5545. 10.1093/nar/gkh894 15486203 PMC524302

[46] Vogel C, Marcotte EM. Insights into the regulation of protein abundance from proteomic and transcriptomic analyses. Nat Rev Genet. 2012;13: 227–232. 10.1038/nrg3185 22411467 PMC3654667

[47] Le Roch KG, Johnson JR, Florens L, Zhou Y, Santrosyan A, Grainger M, et al Global analysis of transcript and protein levels across the *Plasmodium falciparum* life cycle. Genome Res. 2004;14: 2308–2318. 10.1101/gr.2523904 15520293 PMC525690

[48] Legube G, Trouche D. Regulating histone acetyltransferases and deacetylases. EMBO Rep. 2003;4: 944–947. 10.1038/sj.embor.embor941 14528264 PMC1326399

[49] Bömke C, Tudzynski B. Diversity, regulation, and evolution of the gibberellin biosynthetic pathway in fungi compared to plants and bacteria. Phytochemistry. 2009;70: 1876–1893. 10.1016/j.phytochem.2009.05.020 19560174

[50] Proctor RH, Desjardins AE, Plattner RD, Hohn TM. A polyketide synthase gene required for biosynthesis of fumonisin mycotoxins in *Gibberella fujikuroi* mating population A. Fungal Genetics and Biology. 1999;27: 100–112. 10.1006/fgbi.1999.1141 10413619

[51] Seo J-A, Proctor RH, Plattner RD. Characterization of four clustered and coregulated genes associated with fumonisin biosynthesis in *Fusarium verticillioides*. Fungal Genetics and Biology. 2001;34: 155–165. 10.1006/fgbi.2001.1299 11728154

[52] Arst HN, Cove DJ. Nitrogen metabolite repression in *Aspergillus nidulans*. Mol Gen Genet. 1973;126: 111–141. 4591376 10.1007/BF00330988

[53] Marzluf GA. Genetic regulation of nitrogen metabolism in the fungi. Microbiol Mol Biol Rev. 1997;61: 17–32. 9106362 10.1128/mmbr.61.1.17-32.1997 PMC232598

[54] Ment D, Alkan N, Luria N, Bi F-C, Reuveni E, Fluhr R, et al A Role of AREB in the Regulation of PACC-Dependent Acid-Expressed-Genes and Pathogenicity of Colletotrichum gloeosporioides. Mol Plant Microbe Interact. 2015;28: 154–166. 10.1094/MPMI-09-14-0252-R 25317668

[55] Marroquin-Guzman M, Wilson RA. GATA-Dependent Glutaminolysis Drives Appressorium Formation in Magnaporthe oryzae by Suppressing TOR Inhibition of cAMP/PKA Signaling. PLoS Pathog. 2015;11. 10.1371/journal.ppat.1004851 PMC4406744 25901357

[56] Krappmann S, Braus GH. Nitrogen metabolism of Aspergillus and its role in pathogenicity. Med Mycol. 2005;43 Suppl 1: S31–40. 16110790 10.1080/13693780400024271

[57] Magasanik B, Kaiser CA. Nitrogen regulation in *Saccharomyces cerevisiae*. Gene. 2002;290: 1–18. 12062797 10.1016/s0378-1119(02)00558-9

[58] Georis I, Feller A, Tate JJ, Cooper TG, Dubois E. Nitrogen catabolite repression-sensitive transcription as a readout of TOR pathway regulation: the genetic background, reporter gene and GATA factor assayed determine the outcomes. Genetics. 2009;181: 861–874. 10.1534/genetics.108.099051 19104072 PMC2651060

[59] Letting DL, Rakowski C, Weiss MJ, Blobel GA. Formation of a tissue-specific histone acetylation pattern by the hematopoietic transcription factor GATA-1. Mol Cell Biol. 2003;23: 1334–1340. 10.1128/MCB.23.4.1334-1340.2003 12556492 PMC141148

[60] López-Berges MS, Schäfer K, Hera C, Di Pietro A. Combinatorial function of *velvet* and AreA in transcriptional regulation of nitrate utilization and secondary metabolism. Fungal Genet Biol. 2014;62: 78–84. 10.1016/j.fgb.2013.11.002 24240057

[61] Pontecorvo G, Roper JA, Hemmons LM, Macdonald KD, Bufton AWJ. The genetics of *Aspergillus nidulans*. Adv Genet. 1953;5: 141–238. 13040135 10.1016/s0065-2660(08)60408-3

[62] Wiemann P, Albermann S, Niehaus E-M, Studt L, von Bargen KW, Brock NL, et al The Sfp-type 4’-phosphopantetheinyl transferase Ppt1 of *Fusarium fujikuroi* controls development, secondary metabolism and pathogenicity. PLoS ONE. 2012;7: e37519 10.1371/journal.pone.0037519 22662164 PMC3360786

[63] Geissmann TA, Verbiscar AJ, Phinney BO, Cragg G. Studies on the biosynthesis of gibberellins from (−)-kaurenoic acid in cultures of *Gibberella Fujikuroi*. Phytochemistry. 1966;5: 933–947.

[64] Winston F, Dollard C, Ricupero-Hovasse SL. Construction of a set of convenient *Saccharomyces cerevisiae* strains that are isogenic to S288C. Yeast. 1995;11: 53–55. 10.1002/yea.320110107 7762301

[65] Colot HV, Park G, Turner GE, Ringelberg C, Crew CM, Litvinkova L, et al A high-throughput gene knockout procedure for *Neurospora* reveals functions for multiple transcription factors. Proc Natl Acad Sci USA. 2006;103: 10352–10357. 10.1073/pnas.0601456103 16801547 PMC1482798

[66] Schumacher J. Tools for *Botrytis cinerea*: New expression vectors make the gray mold fungus more accessible to cell biology approaches. Fungal Genet Biol. 2012;49: 483–497. 10.1016/j.fgb.2012.03.005 22503771

[67] Sambrook J, Fritsch EF, Maniatis T. Molecular cloning: a laboratory manual. 2nd edn New York: Cold Spring Harbor Laboratory: Cold Spring Harbor; 1989.

[68] Pöggeler S, Masloff S, Hoff B, Mayrhofer S, Kück U. Versatile EGFP reporter plasmids for cellular localization of recombinant gene products in filamentous fungi. Curr Genet. 2003;43: 54–61. 10.1007/s00294-003-0370-y 12684845

[69] Staben C, Jensen B, Singer M, Pollock J, Schechtman M, Kinsey J. Use of a bacterial hygromycin B resistance gene as a dominant selectable marker in *Neurospora crassa* transformation. Fungal Genet Newsl. 1989;36: 79–81.

[70] Tudzynski B, Mende K, Weltring KM, Kinghorn JR, Unkles SE. The *Gibberella fujikuroi niaD* gene encoding nitrate reductase: isolation, sequence, homologous transformation and electrophoretic karyotype location. Microbiology (Reading, Engl). 1996;142 (Pt 3): 533–539. 10.1099/13500872-142-3-533 8868428

[71] Church GM, Gilbert W. Genomic sequencing. Proc Natl Acad Sci USA. 1984;81: 1991–1995. 6326095 10.1073/pnas.81.7.1991 PMC345422

[72] Fisher RA. On the Interpretation of x2 from Contingency Tables, and the Calculation of P. Journal of the Royal Statistical Society. 1922;85: 87–94.

[73] Yates F. The Analysis of Multiple Classifications with Unequal Numbers in the Different Classes. Journal of the American Statistical Association. 1934;29: 51–66.

[74] Gacek-Matthews A, Noble LM, Gruber C, Berger H, Sulyok M, Marcos AT, et al KdmA, a histone H3 demethylase with bipartite function, differentially regulates primary and secondary metabolism in *Aspergillus nidulans*. Molecular Microbiology. 2015;96: 839–860. 10.1111/mmi.12977 25712266 PMC4949671

[75] Todd RB, Fraser JA, Wong KH, Davis MA, Hynes MJ. Nuclear accumulation of the GATA factor AreA in response to complete nitrogen starvation by regulation of nuclear export. Eukaryotic Cell. 2005;4: 1646–1653. 10.1128/EC.4.10.1646-1653.2005 16215172 PMC1265900

[76] Wiśniewski JR, Zielinska DF, Mann M. Comparison of ultrafiltration units for proteomic and N-glycoproteomic analysis by the filter-aided sample preparation method. Anal Biochem. 2011;410: 307–309. 10.1016/j.ab.2010.12.004 21144814

[77] Bergner SV, Scholz M, Trompelt K, Barth J, Gäbelein P, Steinbeck J, et al STATE TRANSITION7-dependent phosphorylation is modulated by changing environmental conditions, and its absence triggers remodeling of photosynthetic protein complexes. Plant Physiol. 2015;168: 615–634. 10.1104/pp.15.00072 25858915 PMC4453777

[78] Kalli A, Hess S. Effect of mass spectrometric parameters on peptide and protein identification rates for shotgun proteomic experiments on an LTQ-orbitrap mass analyzer. Proteomics. 2012;12: 21–31. 10.1002/pmic.201100464 22065615

[79] Martens L, Chambers M, Sturm M, Kessner D, Levander F, Shofstahl J, et al mzML--a community standard for mass spectrometry data. Mol Cell Proteomics. 2011;10: R110.000133. 10.1074/mcp.R110.000133 PMC3013463 20716697

[80] Deutsch E. mzML: a single, unifying data format for mass spectrometer output. Proteomics. 2008;8: 2776–2777. 10.1002/pmic.200890049 18655045

[81] Kessner D, Chambers M, Burke R, Agus D, Mallick P. ProteoWizard: open source software for rapid proteomics tools development. Bioinformatics. 2008;24: 2534–2536. 10.1093/bioinformatics/btn323 18606607 PMC2732273

[82] Bald T, Barth J, Niehues A, Specht M, Hippler M, Fufezan C. pymzML--Python module for high-throughput bioinformatics on mass spectrometry data. Bioinformatics. 2012;28: 1052–1053. 10.1093/bioinformatics/bts066 22302572

[83] Kremer LPM, Leufken J, Oyunchimeg P, Schulze S, Fufezan C. Ursgal, universal python module combining common bottom-up proteomics tools for large-scale analysis. J Proteome Res. 2016;15: 788–794. 10.1021/acs.jproteome.5b00860 26709623

[84] Geer LY, Markey SP, Kowalak JA, Wagner L, Xu M, Maynard DM, et al Open mass spectrometry search algorithm. J Proteome Res. 2004;3: 958–964. 10.1021/pr0499491 15473683

[85] Craig R, Beavis RC. TANDEM: matching proteins with tandem mass spectra. Bioinformatics. 2004;20: 1466–1467. 10.1093/bioinformatics/bth092 14976030

[86] Kim S, Mischerikow N, Bandeira N, Navarro JD, Wich L, Mohammed S, et al The generating function of CID, ETD, and CID/ETD pairs of tandem mass spectra: applications to database search. Mol Cell Proteomics. 2010;9: 2840–2852. 10.1074/mcp.M110.003731 20829449 PMC3101864

[87] Barth J, Bergner SV, Jaeger D, Niehues A, Schulze S, Scholz M, et al The interplay of light and oxygen in the reactive oxygen stress response of *Chlamydomonas reinhardtii* dissected by quantitative mass spectrometry. Mol Cell Proteomics. 2014;13: 969–989. 10.1074/mcp.M113.032771 24482124 PMC3977195

[88] Käll L, Canterbury JD, Weston J, Noble WS, MacCoss MJ. Semi-supervised learning for peptide identification from shotgun proteomics datasets. Nat Methods. 2007;4: 923–925. 10.1038/nmeth1113 17952086

[89] Käll L, Storey JD, MacCoss MJ, Noble WS. Assigning significance to peptides identified by tandem mass spectrometry using decoy databases. J Proteome Res. 2008;7: 29–34. 10.1021/pr700600n 18067246

[90] Höhner R, Barth J, Magneschi L, Jaeger D, Niehues A, Bald T, et al The metabolic status drives acclimation of iron deficiency responses in *Chlamydomonas reinhardtii* as revealed by proteomics based hierarchical clustering and reverse genetics. Mol Cell Proteomics. 2013;12: 2774–2790. 10.1074/mcp.M113.029991 23820728 PMC3790290

