# Supplementary material for: Comparative transcriptome and proteome analysis reveals a global impact of the nitrogen regulators AreA and AreB on secondary metabolism in Fusarium fujikuroi
Source: PLoS One. 2017 Apr 25;12(4):e0176194. doi: 10.1371/journal.pone.0176194 (PMC5404775; doi:10.1371/journal.pone.0176194)
Supplement: S6 Table — (DOCX) [file pone.0176194.s006.docx]

**S6 Table** List of primers used in this study.

| **Name** | **Sequence (5‘ – 3‘)** | **Usage** |
| --- | --- | --- |
| 00008_aps6_F | CTCCAGATCATGAGCGCCTCC | Generation of *APF6* probes |
| 00008_aps6_R | CTCGCAATCCGCATCTGGC | Generation of *APF6* probes |
| AreA-seq5 | TCAGGATTCTCATCTGTGGCC | Diagnostic PCR |
| areB-for-neu | CCGATGCAAATGGATTCGACACGACC | Generation of *AREB* probes |
| areB-rev-neu | CTAGAGGCTTCCGAAGCCAGCAATGC | Generation of *AREB* probes |
| AreB-seq3 | AAATGGATTCGACACGACCGG | Diagnostic PCR |
| bik2-F | CTTGAGTCTGATAGAGGCGC | Generation of *BIK2* probes |
| bik2-R | ACGGCGCAGCAGAAAGTGCC | Generation of *BIK2* probes |
| cps/ks-RT-for | GTGTAGCTGGATCATAGCGACACTCCTG | Generation of *CPS/KS* probes |
| cps/ks-RT-rev | CCATTGGCCCTGGCTAAGTTTCCC | Generation of *CPS/KS* probes |
| FF02109-WT-F | TCACGATGGCCTTGGTAATGG | Generation of *FUB5* probes |
| FF02109-WT-R | ACAATGCTGTCATACGAGCCG | Generation of *FUB5* probes |
| fum8_F | AGTGGTGGCAAGATTGTGG | Generation of *FUM8* probes |
| fum8_R | ATCGTCGAGGTATTGCTTCG | Generation of *FUM8* probes |
| ogfp-seqR1 | CGTCTCCCTCACCCTCTCCG | Diagnostic PCR |
| Tam1-F | CCTATCGCCATTGCTCCTAAACC | Generation of *TAMA* probes |
| Tam1-R | TGGCTTCATCAAGGGTTGTACGC | Generation of *TAMA* probes |
