# Supplementary figures and images for: Comparative transcriptome and proteome analysis reveals a global impact of the nitrogen regulators AreA and AreB on secondary metabolism in Fusarium fujikuroi
Source: PLoS One. 2017 Apr 25;12(4):e0176194. doi: 10.1371/journal.pone.0176194 (PMC5404775; doi:10.1371/journal.pone.0176194)

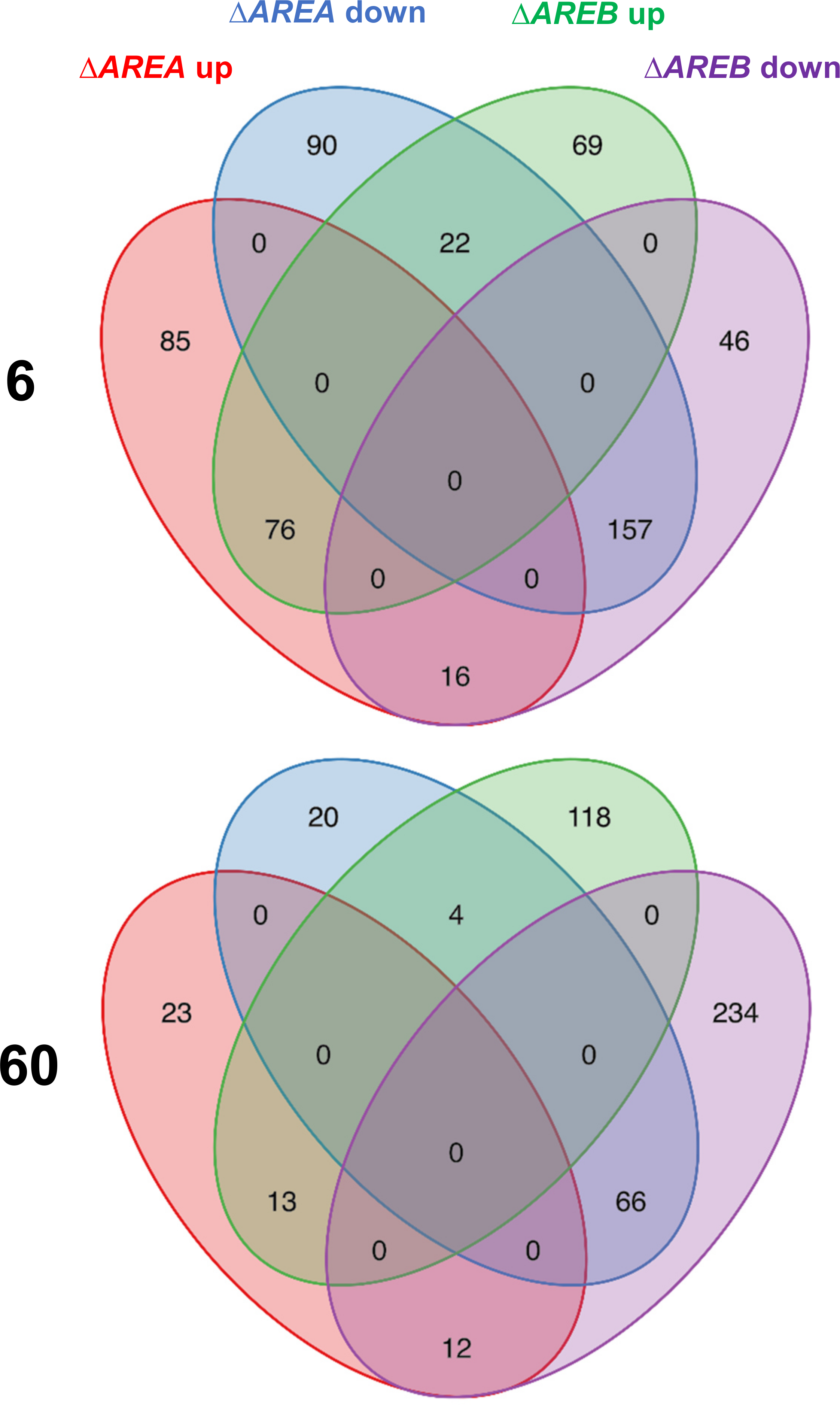

Supplement: S1 Fig — The F. fujikuroi Wt and the ΔAREA and ΔAREB deletion mutants were cultivated for 3 days in ICI liquid cultures with 6 mM (Nitrogen limitation) or 60 mM (nitrogen sufficiency) glutamine as sole nitrogen source. Data is based on proteome analysis. Shown are differentially up-regulated and down-regulated proteins at nitrogen limitation (6) and nitrogen sufficiency (60) in ΔAREA and ΔAREB compared to Wt. (TIF) [file pone.0176194.s007.tif]

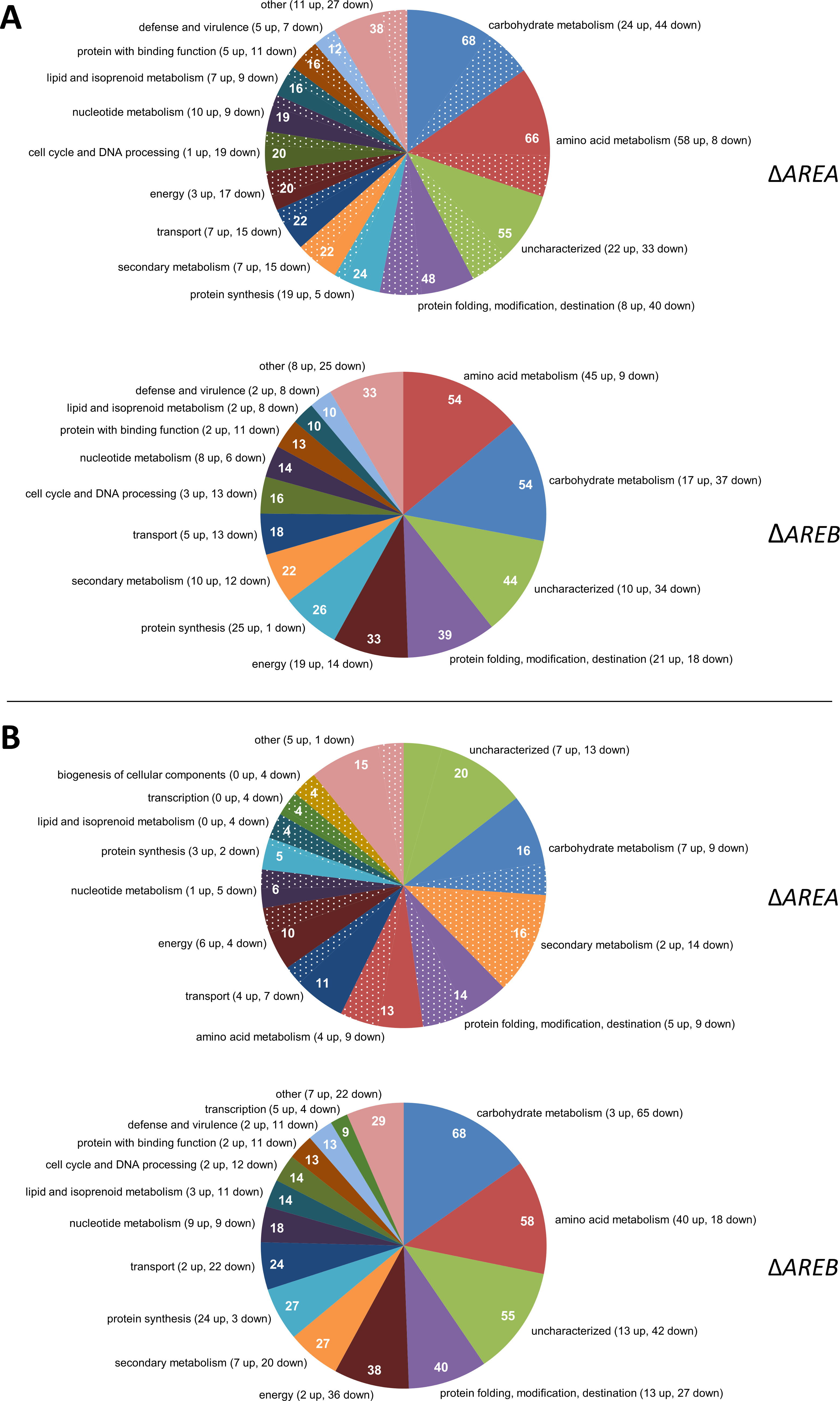

Supplement: S2 Fig — The F. fujikuroi Wt and the ΔAREA and ΔAREB deletion mutants were cultivated for 3 days in ICI liquid cultures with 6 mM (-N) or 60 mM (+N) gln as sole nitrogen source. Data is based on proteome analysis. Differentially regulated proteins in the mutants compared to Wt under respective nitrogen conditions were functionally classified according to their most prominent FunCat category. Stippled areas represent proteins affected in ΔAREA, which are regulated on an equal level (+/- 20%) or stronger in ΔAREB. For these proteins, the regulations might be an indirect effect due to the reduced levels of AreB in the ΔAREA mutant. (TIF) [file pone.0176194.s008.tif]
